# Assessing the importance of primary care diagnoses in the UK Biobank

**DOI:** 10.1007/s10654-023-01095-0

**Published:** 2024-01-16

**Authors:** Lei Clifton, Xiaonan Liu, Jennifer A Collister, Thomas J Littlejohns, Naomi Allen, David J Hunter

**Affiliations:** 1 Nuffield Department of Population Health, University of Oxford, Oxford, UK; 2 UK Biobank Ltd, Stockport, UK; 3 Department of Epidemiology, Harvard TH Chan School of Public Health, Boston, MA USA

**Keywords:** Primary care, General practice (GP), Parkinson’s disease (PD), Type 2 diabetes (T2D), Dementia, UK Biobank

## Abstract

The UK Biobank has made general practitioner (GP) data (censoring date 2016–2017) available for approximately 45% of the cohort, whilst hospital inpatient and death registry (referred to as “HES/Death”) data are available cohort-wide through 2018–2022 depending on whether the data comes from England, Wales or Scotland. We assessed the importance of case ascertainment via different data sources in UKB for three diseases that are usually first diagnosed in primary care: Parkinson’s disease (PD), type 2 diabetes (T2D), and all-cause dementia. Including GP data at least doubled the number of incident cases in the subset of the cohort with primary care data (e.g. from 619 to 1390 for dementia). Among the 786 dementia cases that were only captured in the GP data before the GP censoring date, only 421 (54%) were subsequently recorded in HES. Therefore, estimates of the absolute incidence or risk-stratified incidence are misleadingly low when based only on the HES/Death data. For incident cases present in both HES/Death and GP data during the full follow-up period (i.e. until the HES censoring date), the median time difference between an incident diagnosis of dementia being recorded in GP and HES/Death was 2.25 years (i.e. recorded 2.25 years earlier in the GP records). Similar lag periods were also observed for PD (median 2.31 years earlier) and T2D (median 2.82 years earlier). For participants with an incident GP diagnosis, only 65.6% of dementia cases, 69.0% of PD cases, and 58.5% of T2D cases had their diagnosis recorded in HES/Death within 7 years since GP diagnosis. The effect estimates (hazard ratios, HR) of established risk factors for the three health outcomes mostly remain in the same direction and with a similar strength of association when cases are ascertained either using HES only or further adding GP data. The confidence intervals of the HR became narrower when adding GP data, due to the increased statistical power from the additional cases. In conclusion, it is desirable to extend both the coverage and follow-up period of GP data to allow researchers to maximise case ascertainment of chronic health conditions in the UK.

**Supplementary Information:**

The online version contains supplementary material available at 10.1007/s10654-023-01095-0.

## Introduction

The UK Biobank (UKB) is an ongoing population-based prospective cohort study of approximately 500,000 participants recruited across England, Scotland and Wales between 2006 and 2010 [1]. The resource is widely used by researchers across the globe for conducting health-related research, in particular for identifying novel risk factors associations with a range of diseases that mostly occur at middle and older ages.

In order to enable longitudinal analyses, UKB performs ongoing linkage to a range of electronic health administrative datasets, which currently includes hospital inpatient records, cancer and death registry data. These datasets represent the main source of health outcome ascertainment for a range of different diseases and are regularly updated by UKB. For a subset of the cohort (45%), primary care data are available up until 2016–2017.

Hospital admissions are recorded in the Hospital Episode Statistics for England (HES), Scottish Morbidity Record (SMR) and Patient Episode Database for Wales (PEDW) for England, Scotland and Wales, respectively [1]. We will collectively refer to all three sources of hospital inpatient data as “HES” data. Death records in England and Wales are provided by NHS England, and Scotland by NHS Central Register, National Records of Scotland. The HES diagnoses include the main reason for hospital admission, as well as other underlying health conditions. Thus, ambulatory conditions that often do not initially require hospitalisation and are typically diagnosed in primary care (i.e. GP) data, may (or may not) be subsequently recorded in hospital inpatient records depending on whether patients are admitted for another condition and whether the ambulatory condition is recorded in the inpatient records.

The aim of this study is to determine the added-value of incorporating GP data to that of HES and death data when ascertaining cases of Parkinson’s disease (PD), type 2 diabetes (T2D), and dementia for epidemiologic analyses. These conditions were selected because they are likely to be initially diagnosed in primary care, prior to any hospital record. Furthermore, as they are usually managed within primary care, their documentation in the corresponding HES records is usually not the primary reason for the hospitalisation.

## Methods

### Risk factors

Established risk factors for each disease were identified from the literature [2–6]. We used the risk factors that are assessed in UKB, and are applicable to the UK (full detail in Supplementary Tables 1–2, and Supplementary Fig. 1). We kept the derivation and categorisation of risk factors consistent across the diseases wherever possible. For example, we used BMI “underweight/normal, overweight, and obese” consistently.

### Outcome definitions

We used the “code lists for algorithmically-defined outcomes” (UKB Resource 594) developed by the UKB team to identify Parkinson’s disease and dementia cases. These code lists contain diagnostic and medication codes for PD, and diagnostic (no medication) codes for dementia. These diagnostic codes include UKB self-report, ICD-9, ICD-10, and Read codes. T2D is not currently included in these code lists, and we instead used clinical codes as reported from the existing literature [7].

### Study populations

We applied the following exclusion criteria for each of the three diseases:

- Age outside of the UKB enrolment criterion of 40 to 69 years.
- Those without GP data (i.e. we only analysed the ~ 45% UKB participants who had GP data available).
- Prevalent cases of the disease of interest.

For dementia, we further excluded individuals younger than 60 years at baseline, to ensure the sample was restricted to those most at risk of developing dementia during the follow-up period. We also further excluded participants with missing APOE e4 carrier status.

For diabetes, we excluded both prevalent Type 1 diabetes (T1D) and T2D cases at baseline (i.e. UKB enrolment) [8]. The “prevalence algorithm 1” by Eastwood et al. [9] and hospital inpatient records were used to identify prevalent type 1 or type 2 diabetes at baseline.

**Fig. 1:**
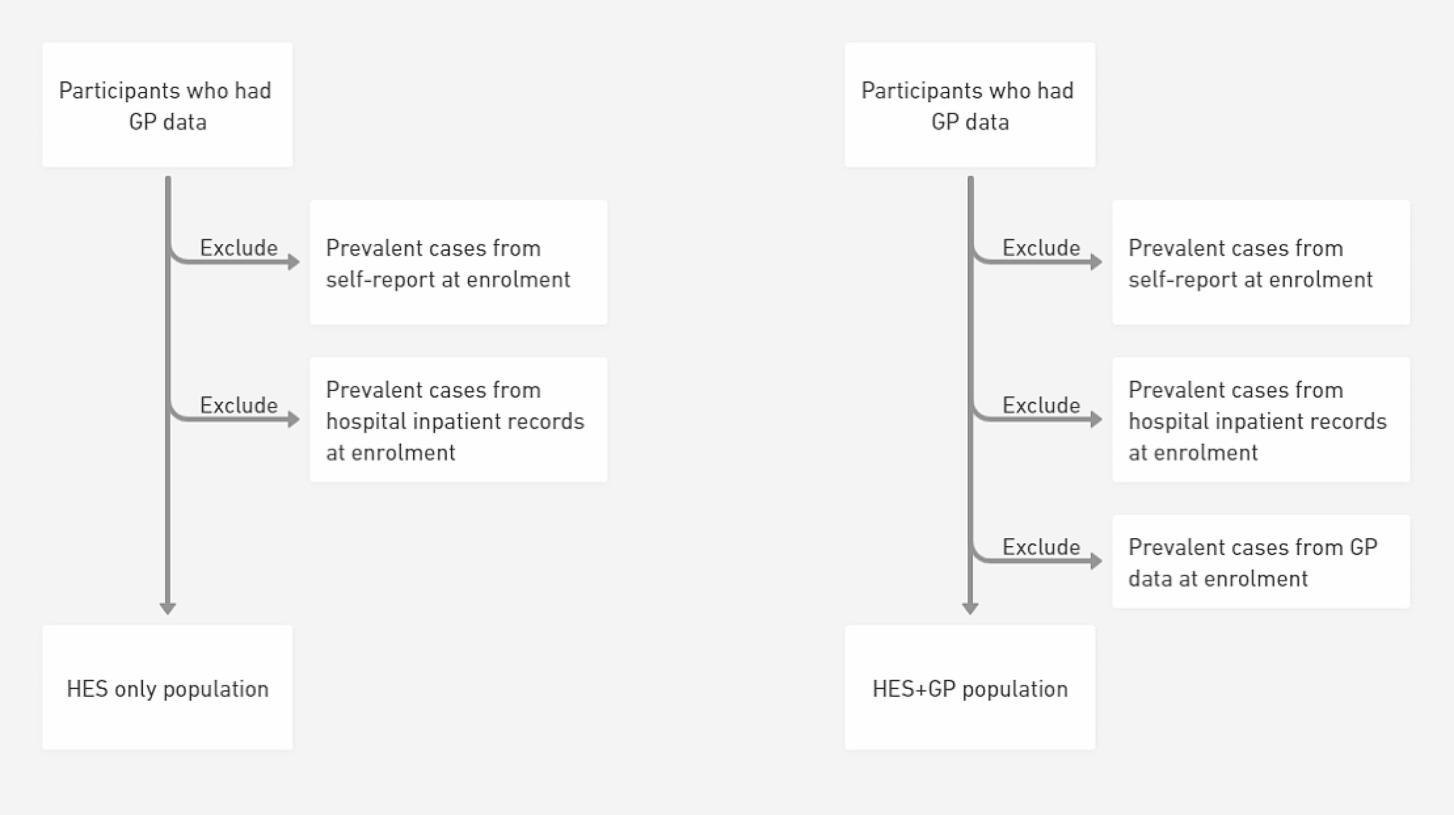
Conceptual diagram illustrating the difference between the “HES only” and “HES + GP” populations. Our study is based on the ~ 45% UKB participants whose GP data are available; i.e. we do not consider those without GP data

The differences in sample size between two study populations (“HES only” and “HES + GP”) for each of the three diseases are illustrated in Fig. 1.

- For the “HES only” population, we used (i) self-report (diagnoses and medications) UKB data at enrolment date, and (ii) hospital inpatient data prior to or at enrolment, to identify prevalent cases.
- For the “HES + GP” population, we further incorporated (iii) GP data (diagnoses and medications) to identify prevalent cases. This study population is slightly smaller than the “HES only” population, since some prevalent cases may be present only in GP data, but not in the self-reported UKB data or hospital inpatient data.

The definition of prevalent and incident cases are shown in Supplementary Table 3. We note that *prevalent* cases are excluded to define the study population, whereas *incident* cases are for obtaining estimates of the incidence of the disease.

Incident cases were ascertained longitudinally using record-level hospital inpatient data and death registry (hereafter referred to as “HES/Death”) for both populations. GP diagnosis data were used additionally for the “HES + GP” population (Supplementary Table 3).

### Censoring approach

To compare incidences across the same follow-up period between the “HES only” and “HES + GP” populations, we applied the GP administrative censoring date (2016–2017, hereafter referred to as “GP censoring date”) for both populations [10]. Therefore, the only difference between the two populations is that the “HES + GP” population have GP data as an additional data source, thereby enabling a direct comparison of the added value of including GP data for case ascertainment purposes.

However, the “GP censoring date” (2016–2017) was earlier than the “HES censoring date” (2018–2022, depending on whether the data come from England, Wales, or Scotland), as illustrated in Supplementary Fig. 11. This means that our follow-up period for the primary analyses (e.g. median 7.0 years in “HES only” population for dementia) is shorter than what researchers would typically use if they are relying on hospital and death data (i.e. without GP data) for case ascertainment. Further details on different censoring approaches can be found in Supplementary Table 6.

The follow-up time for each participant was calculated as the number of years from the date of UKB enrolment until the earliest of the following dates:

- First occurrence of the health condition (diagnosis or death).
- Death from causes other than the outcome of interest.
- Loss to follow-up (e.g. emigration or withdrawal from the study).
- GP censoring date: 2016 in England (TPP supplier) and 2017 in England (Vision supplier), Scotland and Wales (Supplementary Fig. 11).
- Final deduction date from GP data (i.e. the date a participant was recorded as leaving a GP). Approximately 3% of participants had conflicting records showing them joining two or more GP on the same day; we resolved this discrepancy by choosing the most recent record.

### Quantifying differences

To quantify the difference between the “HES only” and “HES + GP” populations for each disease, we first plotted the cumulative incidence by family history - a risk factor shared across all three diseases. To quantify the difference in estimated effect of risk factors with outcome between the two populations, we constructed respective Cox models to obtain the hazard ratios (HR) for comparison. Missing data were replaced by multiple imputation (10 imputed datasets) under the assumption of missing at random using the *mice* package. The missing percentage of all variables are reported in Supplementary Tables 4–5.

We presented the ratio of HR (RHR) to provide a direct comparison of the HR obtained from the two respective populations. Bootstrap inference with multiple imputation [11] was used to calculate the confidence intervals (CI) of the RHR [12] (Supplementary Fig. 16). Statistical tests were two-tailed at a 5% significance level.

## Results

### Baseline characteristics

After applying the exclusion criteria to the 502,368 UKB participants, approximately 221,000, 210,000 and 90,700 participants were available for analysis for PD, T2D and dementia, respectively, for both “HES only” and “HES + GP” populations (detailed flow charts in Supplementary Figs. 2–4).

Table 1 shows that for all three diseases, including GP data at least doubled the number of incident cases compared with those diagnosed when only using HES/Death data (“HES only” population). For example, the number of incident cases for dementia increased from 619 in the “HES only” population to 1390 in the “HES + GP” population. Note that in the “HES + GP” population, cases diagnosed in the GP data prior to baseline were excluded, and therefore the number of cases diagnosed in the HES/Death data will be lower than that in the “HES only” population.

**Table 1:** Incident cases among the “HES only” and “HES + GP” study populations for each disease. The rows show the “number of incident cases / number of participants” and follow-up period. IQR: interquartile range. We note that the “HES only” and “HES + GP” population sizes are slightly different; this is because incident cases in the “HES only” population can become prevalent cases in the “HES + GP” population, where prevalent cases in the GP data were excluded from the “HES + GP” population, as shown in Fig. 1

		Parkinson’s Disease	Type 2 Diabetes	Dementia
HES only	cases / population (%)	377 / 221,167 (0.17%)	3431 / 209,988 (1.63%)	619 / 90,700 (0.68%)
	Median (IQR) follow-up in years	7.1 (6.25, 7.93)	7.1 (6.22, 7.92)	7.0 (6.21, 7.84)
HES + GP	cases / population (%)	740 / 221,041 (0.33%)	7829 / 209,684 (3.73%)	1390 / 90,668 (1.53%)
	Median (IQR) follow-up in years	7.1 (6.25, 7.93)	7.0 (6.16, 7.89)	7.0 (6.19, 7.83)

**Fig. 2:**
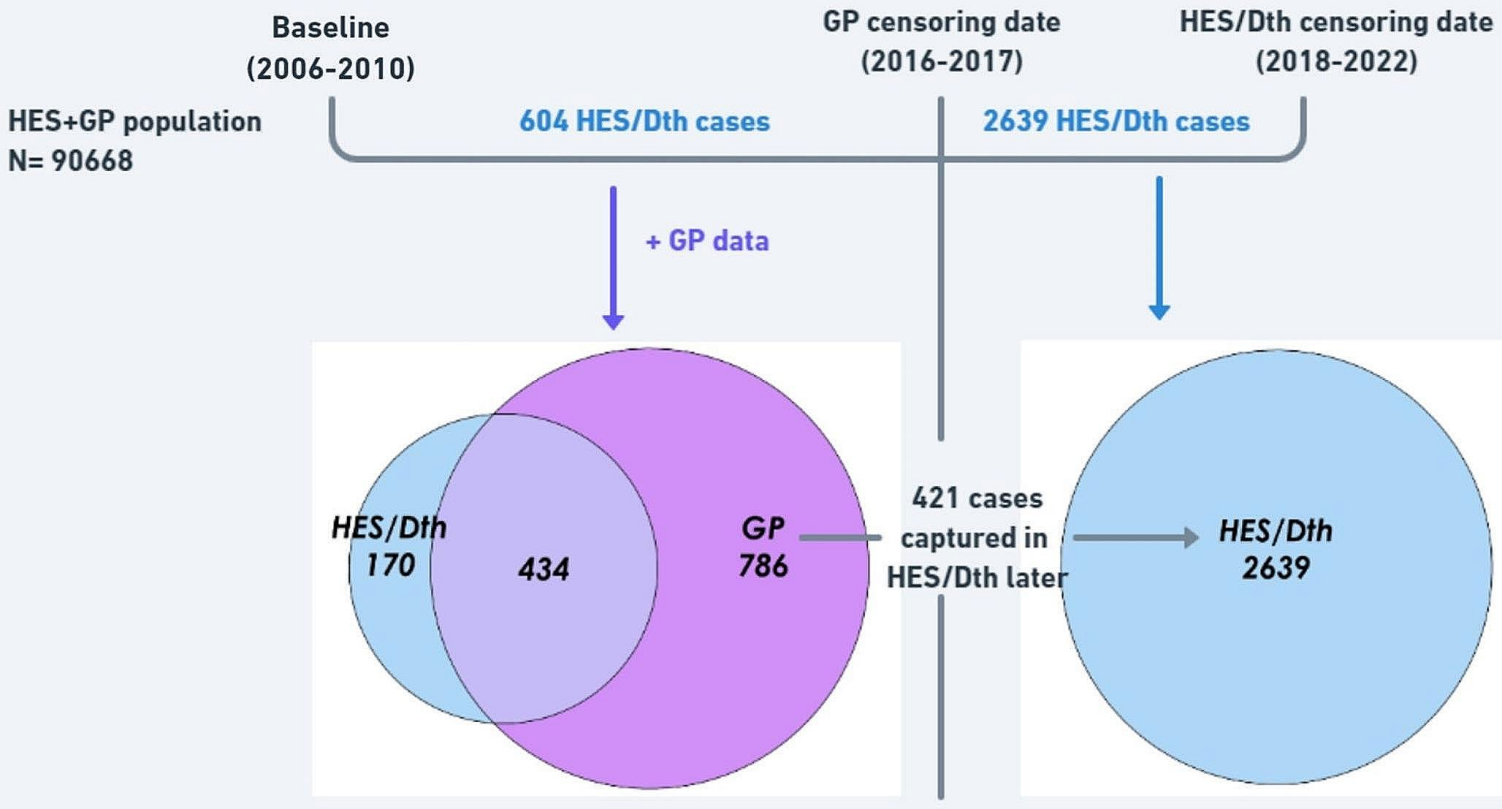
Venn diagram comparing incident cases of dementia from HES/Death and those from GP records in the “HES + GP” population (n = 90,668). Among the 786 cases in GP (but not in HES/Death) data prior to the GP censoring date, 421 appeared in HES/Death later; i.e. 365 (= 786 − 421) cases were unique to the GP data even after allowing for the extended follow-up in the HES/Death data. Please see Sect. 3.4 and Supplementary Table 6 for details on different censoring approaches. Using the HES/Death data beyond the GP censoring date, 2218 (= 2639 − 421) further cases were recorded in the HES/Death data, but we do not know how many appeared in the subsequent GP records due to the lack of these records after 2016–2017. Dth: Death

Figure 2 shows that of the 786 dementia cases (before GP censoring date) in the “HES + GP” population that were initially only recorded in the GP data, 421 appeared later in HES/Death data, after GP censoring date. Similar phenomena were observed for PD and T2D in Table 1 (detailed Venn diagrams in Supplementary Figs. 5, 7, and 9).

Combining the numbers in Table 1; Fig. 2, we can examine among the prevalent cases captured by the GP data but excluded from the “HES + GP” population, how many subsequently appeared in the HES/Death data. Table 1 shows that 32 (= 90,700 − 90,668) individuals from the “HES only” population for dementia were excluded in the “HES + GP” population. Figure 2 shows that 604 dementia cases in the “HES + GP” population were captured in the HES/Death data before the GP censoring date, compared to the 619 dementia cases in the “HES only” population (Table 1). We can therefore conclude that of the 32 individuals identified as prevalent dementia cases in the GP data, only 15 (= 619 − 604) were subsequently captured in the HES/Death data; the remaining were incorrectly regarded as non-cases in the “HES only” population. Similar considerations apply to the Supplementary Figures for PD and T2D.

For incident cases present in both HES/Death and GP data during the full follow-up period (i.e. until the HES censoring date), we plotted histograms (Supplementary Figs. 5, 7, 9) showing the distributions of the time difference (i.e. lag) between diagnosis dates between the two data sources. These median (interquartile range, IQR) time differences in years were 2.31 (0.83, 4.60) for PD, 2.82 (1.07, 5.30) for T2D, and 2.25 (0.76, 4.20) for dementia.

We note that the above represents the latency (i.e. time between a diagnoses being recorded in GP compared with HES/Death data) among those who had records in both GP and HES/Death data. For participants with an incident GP diagnosis, only 65.6% of dementia cases, 69.0% of PD cases, and 58.5% of T2D cases had their initial GP diagnosis recorded in HES/Death within 7 years since GP diagnosis (Supplementary Figs. 6, 8, and 10).

We note that these numbers reflect recorded diagnoses made during the available follow-up period (that differs for each participant). For example, if a participant had a GP diagnosis of dementia in 2016 (i.e. close to GP censoring date), and was followed up for a further 5 years until 2021 (i.e. close to the HES censoring date), this might not be long enough for the diagnosis to be captured in the HES record. In contrast, a participant with an earlier GP diagnosis (e.g. 2010) would have had a longer time period in which their diagnosis could be captured in the HES data.

The baseline characteristics of the “HES only” and “HES + GP” populations are very similar. The overlapping variables of the three diseases for the “HES + GP” population are shown in Table 2. Detailed baseline characteristics of both populations are in Supplementary Tables 4–5.

**Table 2:** Baseline characteristics of the “HES + GP” population for Parkinson’s disease (PD), type 2 diabetes (T2D), and dementia. Family history represents family history of PD, T2D, and dementia

	PD (N = 221,041)	T2D (N = 209,684)	Dementia (N = 90,668)
**Age at enrolment**			
Mean (SD)	56.99 (8.02)	56.83 (8.03)	64.55 (2.81)
Min, Max	40.11, 69.99	40.11, 69.99	60.00, 69.99
**Self-reported ethnicity**			
White	209,643 (94.8%)	199,722 (95.2%)	88,134 (97.2%)
Black	2441 (1.1%)	2187 (1.0%)	403 (0.4%)
S. Asian	3829 (1.7%)	3140 (1.5%)	950 (1.1%)
Mixed	1113 (0.5%)	1056 (0.5%)	202 (0.2%)
Other	2964 (1.3%)	2633 (1.3%)	609 (0.7%)
Missing	1051 (0.5%)	946 (0.5%)	370 (0.4%)
**Gender**			
Female	121,043 (54.8%)	116,750 (55.7%)	47,977 (52.9%)
Male	99,998 (45.2%)	92,934 (44.3%)	42,691 (47.1%)
**Townsend Deprivation Index**			
Mean (SD)	-1.32 (3.04)	-1.38 (3.01)	-1.56 (2.92)
Min, Max	-6.26, 11.00	-6.26, 11.00	-6.26, 10.50
Missing	323 (0.1%)	304 (0.1%)	91 (0.1%)
**Family history**			
No	212,191 (96.0%)	167,165 (79.7%)	76,929 (84.8%)
Yes	8850 (4.0%)	42,519 (20.3%)	13,739 (15.2%)

### Cumulative incidence

**Fig. 3:**
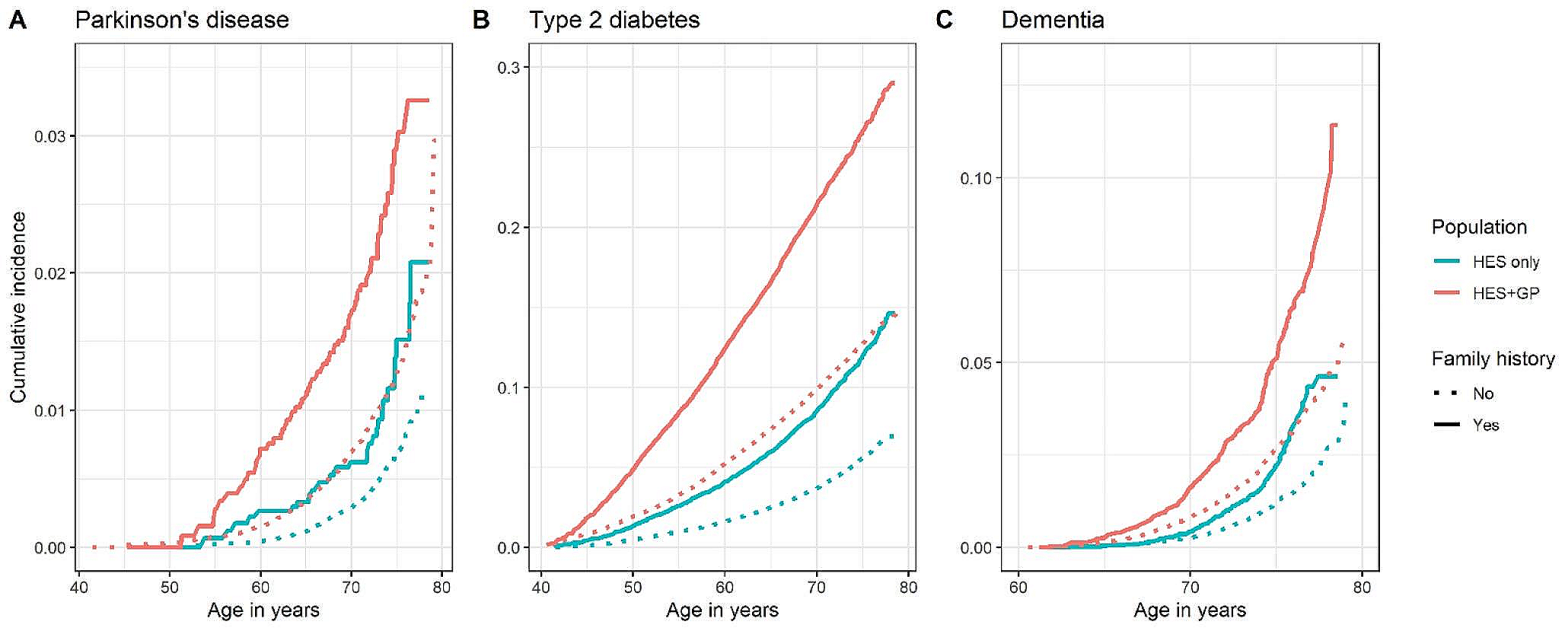
Age specific cumulative incidence plots by family history, for all three diseases. Note that the ranges of the y-axis are different in the three subplots

To illustrate differences in cumulative incidence stratified by a risk factor, we plotted the age-specific cumulative incidence of each disease stratified by family history - a common predictor for all three diseases. Figure 3 shows that for PD and T2D, the additional GP data approximately doubles the number of incident cases across all ages, regardless of family history. This trend is maintained for dementia, but less prominent towards the older age of 75–80 years. These age-specific cumulative incidence plots are overall consistent with the incident cases shown in Table 1.

### Results obtained from Cox models

**Fig. 4:**
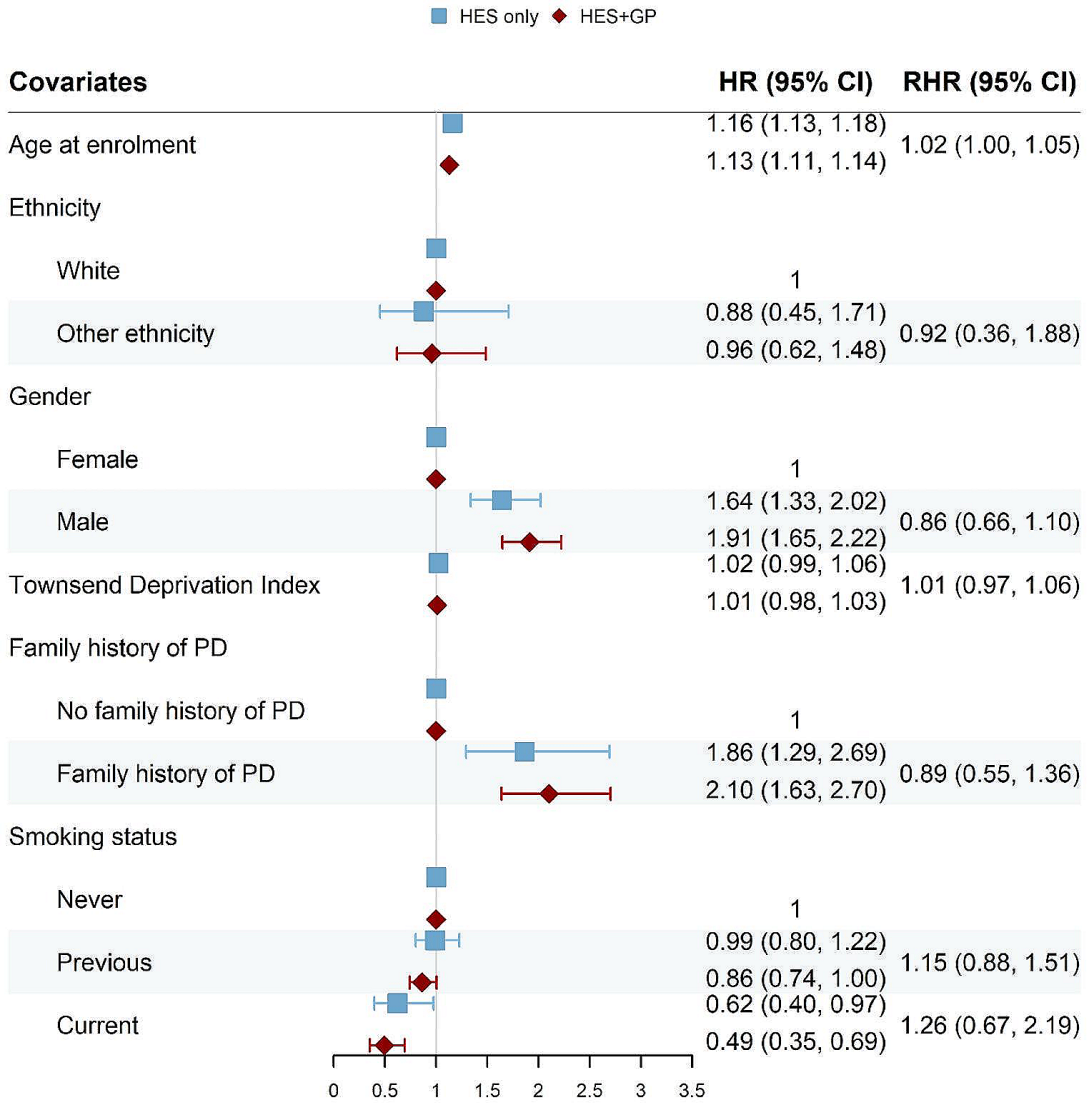
Forest plot showing hazard ratios (HR) obtained from the Cox proportional hazard models for Parkinson’s Disease (PD), using the “HES only” and “HES + GP” populations, respectively. The corresponding ratio of HR (RHR) is shown with its 95% CI obtained from bootstrapping

**Fig. 5:**
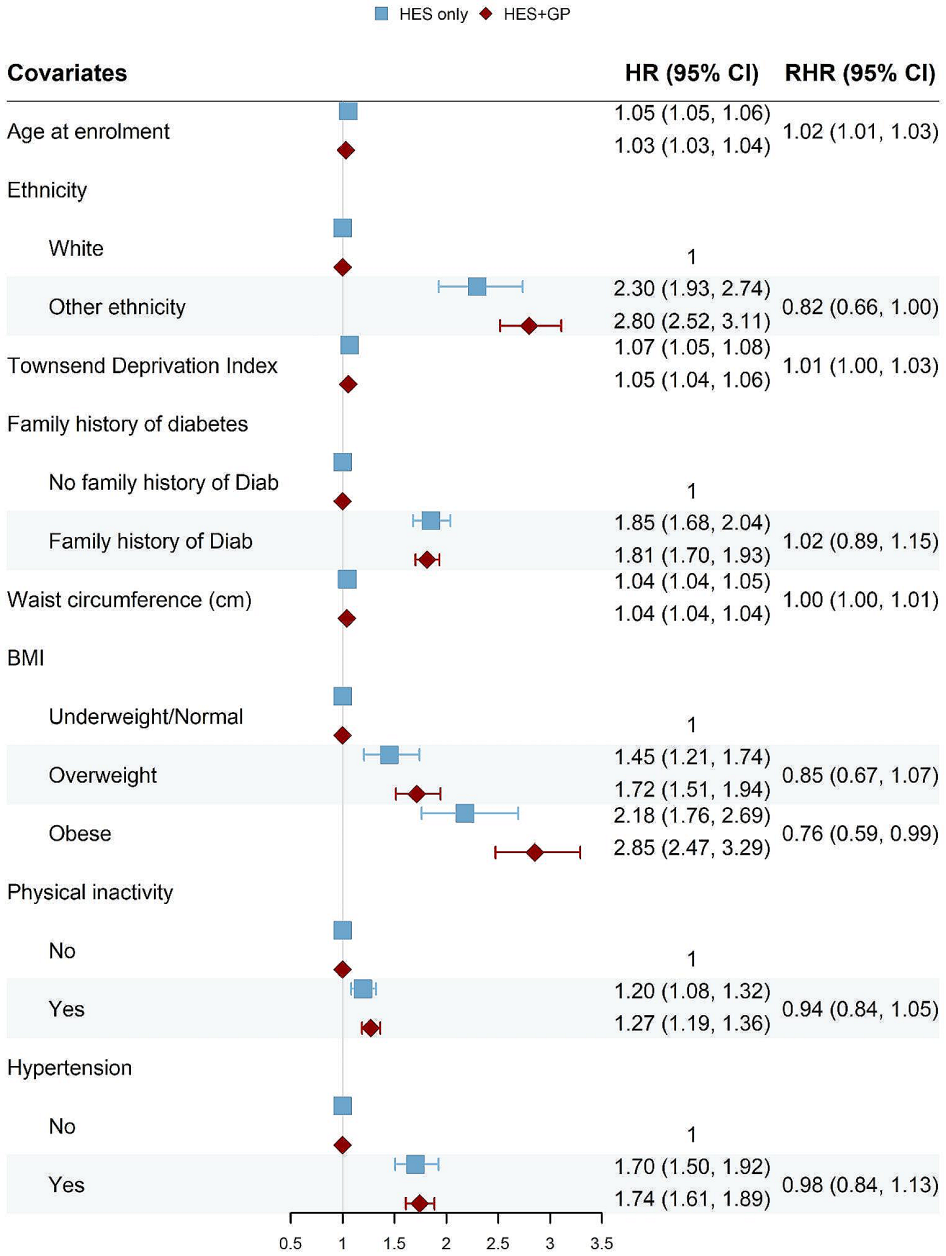
Forrest plot for Type 2 Diabetes (Male only)

**Fig. 6:**
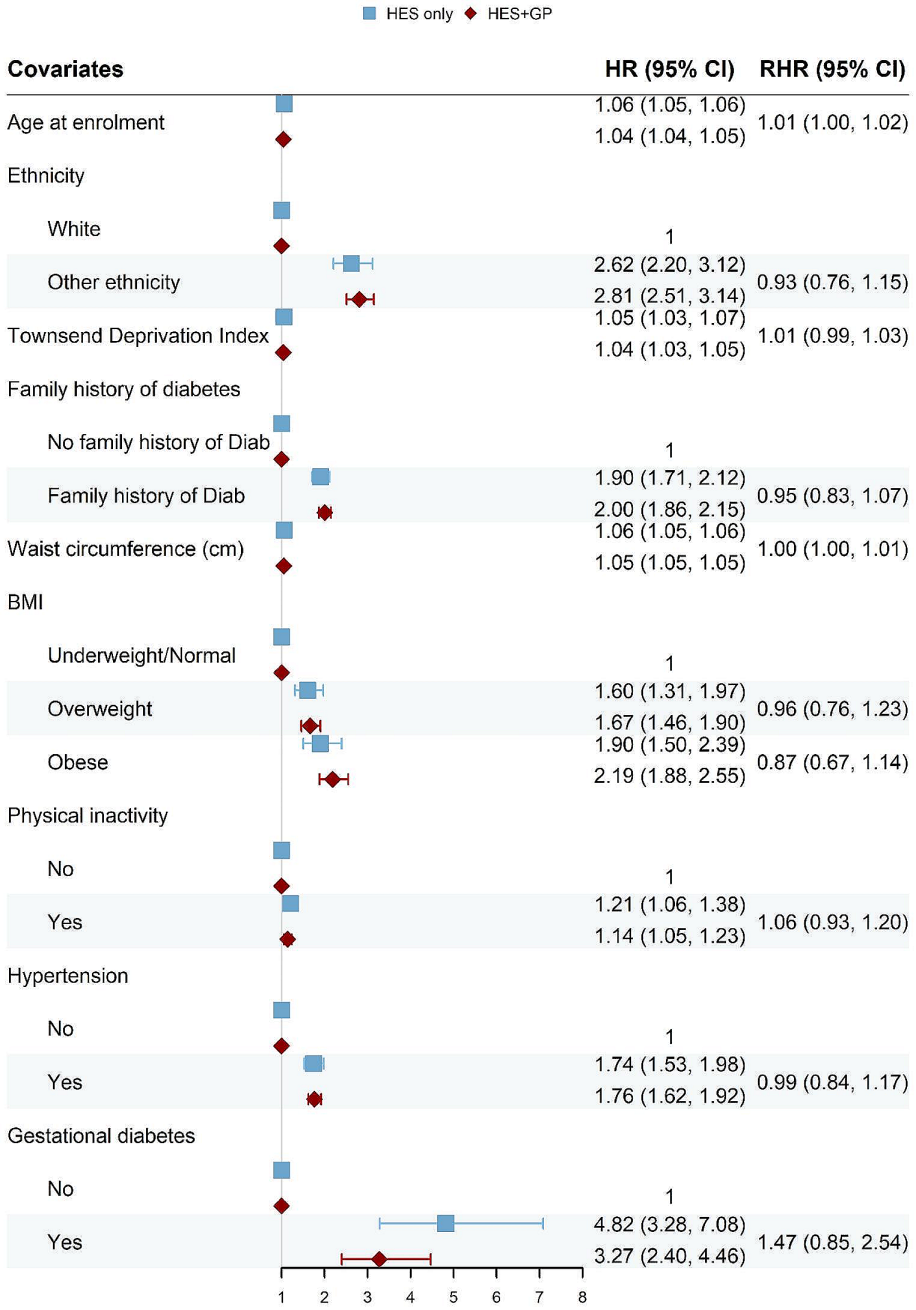
Forrest plot for Type 2 Diabetes (Female only)

**Fig. 7:**
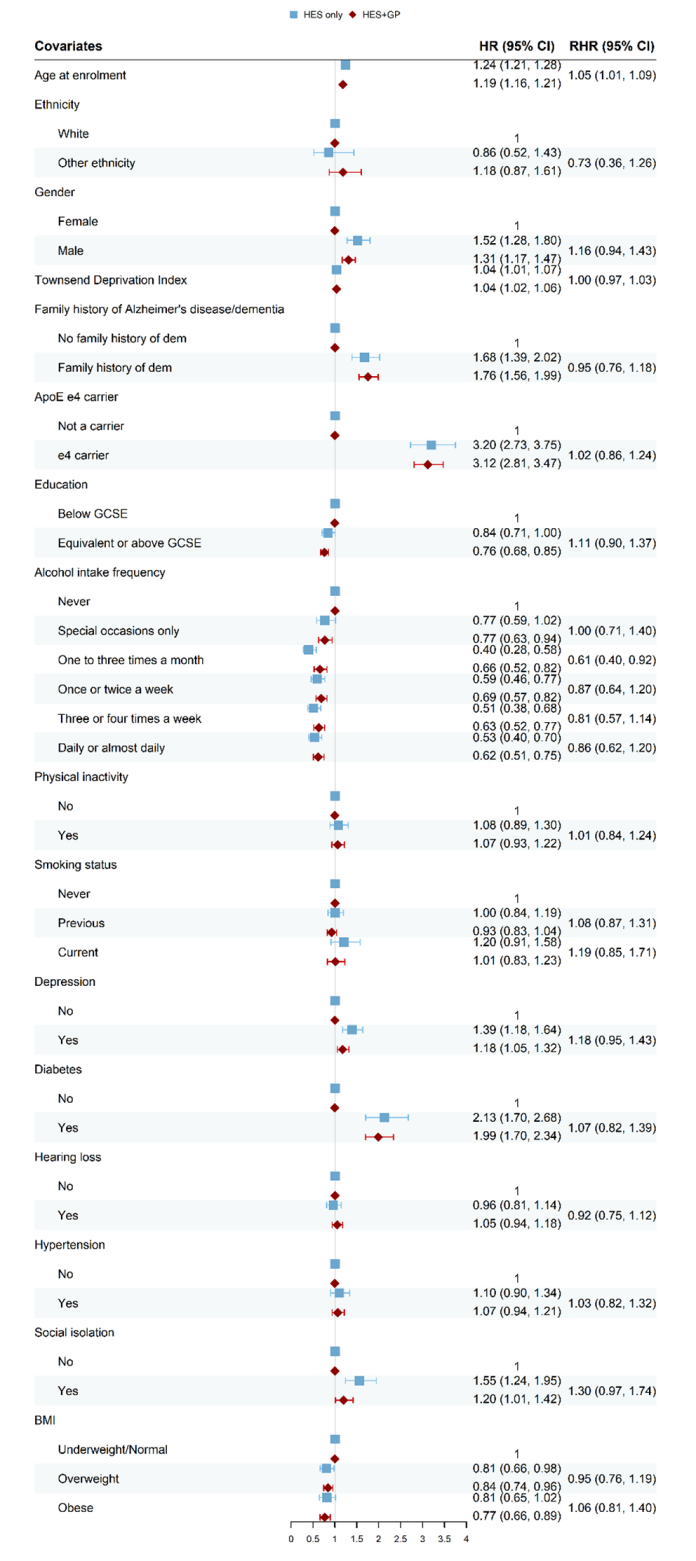
Forest plot for Dementia

We built Cox proportional hazard models for each of the disease outcome defined in Methods. The resulting forest plots (Figs. 4, 5, 6 and 7) show the HR obtained from the “HES only” and “HES + GP” populations, respectively (details in Supplementary Tables 9–12). Similar results were obtained using complete-case analyses (Supplementary Figs. 12–15 and Supplementary Tables 13–16). The HR are largely in the same direction, and of comparable magnitude, indicating the overall agreement between the two populations. The confidence intervals (CI) of the HR obtained from the “HES + GP” populations are narrower than those from the “HES only” population, due to the increased statistical power from the additional incident cases in GP data.

To provide a statistical comparison between the two HR, we calculated the corresponding RHR and used bootstrap to obtain its 95% CI. An RHR < 1 means the “HES only” population yields a smaller HR than the “HES only” population, and vice versa. Among overlapping risk factors, only age had a statistically significant RHR for all three health outcomes by source of case ascertainment.

Our estimated effect of “hearing loss” on dementia in the “HES only” population (HR = 0.96, 95%CI 0.81, 1.14) is in the opposite direction to the existing literature, and therefore we performed additional analyses to examine this inconsistency. Our results (Supplementary Tables 7–8) showed that this is partly due to the short follow-up period in our analyses, in which we censored both populations by the GP censoring date, which is approximately 5 years earlier than the HES censoring date. In an additional sensitivity analysis using a longer follow-up period (i.e. HES censoring date) (Supplementary Tables 7–8), the HR of “hearing loss” in “HES only” population returned to the expected direction (HR = 1.04, 95% CI 0.97, 1.12). These results show that on occasion having limited follow-up period in primary care data may alter conclusions about a risk factor association.

High BMI appears to be inversely associated with incident dementia risk in both “HES only” and “HES + GP” populations. This is most likely caused by reverse causation owing to the short follow-up period of this analysis (we censored both populations by the GP censoring date).

## Discussion

The UKB is increasingly used for the development of risk prediction models, and is one of the few studies incorporating polygenic risks [13]. We show that the age-specific cumulative incidence is more than halved for each of these three diseases when not incorporating GP data - compatible of course, with the fact that more than half of the cases were identified only in the GP data. A similar consideration applies to age at diagnosis which is systematically later in the HES data than those in the GP data, even for the cases that subsequently appear in hospital inpatient data or death registry.

In general, during the period of follow-up for which both primary care and HES/Death data were available, we did not observe large differences in the estimates of established risk factors for these three conditions that are usually first diagnosed in general practice. While limited to three common conditions, these results are reassuring that results based on the full UKB cohort from epidemiological studies of diseases first diagnosed in the community yield comparable estimates for the direction and magnitude of established risk factors for these diseases.

Our purpose was not to replicate the effect estimates of risk factors in the existing literature. Instead, we aimed to quantify the additional benefit of incorporating GP data into UKB for the ascertainment of PD, T2D and dementia. We used the GP censoring date (2016–2017) for both the “HES only” and the “HES + GP” populations to enable direct comparisons of case ascertainment to be made. The extended follow-up data available in the HES/Death data were used to assess the time lag between diagnoses recorded in GP data compared with HES/Death data.

The short follow-up period of this study means that our results are prone to reverse causation, which is a key consideration when investigating associations between risk factors and a disease outcome. This is most noticeable in the estimated association of BMI with dementia, for which being obese appears to be protective for dementia. An individual may experience slow cognitive decline for more than a decade before receiving a definite clinical diagnosis of dementia, and preclinical disease can cause appreciable weight loss during this period [14, 15]. Therefore, being overweight or obese may be associated with seemingly lower risk of dementia due to the short follow-up period (median 7 years) in our study. This further demonstrates the importance of extending the existing follow-up period of the GP data in the UKB cohort, as the current follow-up period is likely insufficient to rule out the bias of reverse causation [16].

GP data were obtained in 2017 from the GP system suppliers who agreed to provide data to the UKB study. These data are largely a representative subset of the cohort as a whole, and we do not anticipate that the different GP system suppliers will have substantial impact on our results.

### Conclusions

Adding GP data in the UKB substantially increased case ascertainment for all three health conditions that are primarily diagnoses and managed in primary care. Including GP data approximately doubled the incident cases, compared with using hospital and death records alone, for all three conditions across ages. Estimates of cumulative incidence of these diseases in risk prediction algorithms will be misleading, if GP data are not included. Our results are largely reassuring that the main established risk factors for these three diseases are apparent, with and without the primary care outcomes being included.

Access to the primary care data enabled more precise estimate of the risk factor-outcome associations for these three diseases, compared with that obtained using only the HES/Death data. However, the relatively early GP censoring date (compared with the HES/Death censoring date for the full cohort) yielded a short follow-up period, and hence limited the number of incident cases available for analysis. The availability of comprehensive cohort-wide primary care data to authorised researchers, is thus highly desirable to enhance the value of epidemiological research using UKB.

### Electronic supplementary material

Below is the link to the electronic supplementary material.

Supplementary Material 1

## Data Availability

This research has been conducted using the UK Biobank Resource under Application Number 33952. Requests to access the data should be made via application directly to the UK Biobank, https://www.ukbiobank.ac.uk.

## Code availability

The code used for analyses are available at https://github.com/xiaonanl1996/HESvsGP.

## Declarations

### Transparency statement

The lead author affirms that this manuscript is an honest, accurate, and transparent account of the study being reported; that no important aspects of the study have been omitted; and that any discrepancies from the study as planned have been explained.

### Patient and community involvement

The analyses presented here are based on existing data from the UK Biobank cohort study, and the authors were not involved in participant recruitment. To the best of our knowledge, no patients were explicitly engaged in the design or implementation of the UK Biobank study. No patients were asked to advise on interpretation or writing these results. Results from UK Biobank are routinely disseminated to study participants via the study website and social media outlets.
