# Supplementary material for: Assessing the importance of primary care diagnoses in the UK Biobank
Source: Eur J Epidemiol. 2024 Jan 16;39(2):219–29. doi: 10.1007/s10654-023-01095-0 (PMC10904436; doi:10.1007/s10654-023-01095-0)
Supplement: Supplementary file 1 — Supplementary Material 1 [file 10654_2023_1095_MOESM1_ESM.docx]

**Assessing the importance of primary care diagnoses in the UK Biobank**

**Supplementary materials**

**Lei Clifton^1*^, Xiaonan Liu^1^, Jennifer A Collister^1^, Thomas J Littlejohns^1^, Naomi Allen^1,2^, David J Hunter^1,3^**

1. Nuffield Department of Population Health, University of Oxford, Oxford, UK

2. UK Biobank Ltd, Stockport, UK

3. Department of Epidemiology, Harvard TH Chan School of Public Health, Boston, MA, USA

* Corresponding Author

Email: lei.clifton@ndph.ox.ac.uk

ORCID: 0000-0001-5595-8468

# Methods

The risk factors of each disease are identified from the existing literature, summary below in Supplementary Table 1.

Supplementary Table 1. Risk factors for Parkinson’s disease, type 2 diabetes, and dementia. Column “Risk factors” includes all the risk factors across the literature in column “Sources”, regardless of whether they are applicable to the UK. We then chose the risk factors suitable for the UKB to be included in this study. For example, we did not include “history of constipation, living in rural area, drinking well water, farming, exposure to pesticides, head injury” when investigating Parkinson’s disease in this study.

| Disease | Risk factors | Sources |
| --- | --- | --- |
| Parkinson’s disease (PD) | Age, sex, ethnicity, family history of PD/tremor, smoking, history of constipation, living in rural area, drinking well water, farming, [exposure to pesticides](https://biobank.ndph.ox.ac.uk/showcase/field.cgi?id=22614), head injury. | [Mayo clinic](https://www.mayoclinic.org/diseases-conditions/parkinsons-disease/symptoms-causes/syc-20376055); [Noyce2012](https://onlinelibrary.wiley.com/doi/full/10.1002/ana.23687); [Hirsch2016](https://www.karger.com/Article/Abstract/445751); [Jacobs2020](https://jnnp.bmj.com/content/91/10/1046.abstract) (UKB-analysis); [Breckenridge2016](https://journals.plos.org/plosone/article?id=10.1371/journal.pone.0151841); [Bloem2021](https://www.thelancet.com/journals/lancet/article/PIIS0140-6736(21)00218-X/fulltext) |
| Type 2 diabetes (T2D) | Age, sex, ethnicity, family history, smoking, waist circumference, BMI, inactivity, metabolic syndrome*, gestational diabetes, heart attack/stroke, Polycystic Ovary Syndrome (PCOS), mental health, sedentary lifestyle, alcohol, sleep, diet, medication**, Low birth weight for gestational age. | [Diabetes UK](https://www.diabetes.org.uk/preventing-type-2-diabetes/diabetes-risk-factors); [NICE](https://cks.nice.org.uk/topics/diabetes-type-2/background-information/risk-factors/); [Xu2022](https://alzres.biomedcentral.com/articles/10.1186/s13195-022-01137-x) |
| Dementia | Age, sex, ethnicity, family history, APOE gene, less education, low social contact, hypertension, hearing impairment, depression, diabetes, traumatic brain injury, smoking, obesity, physical inactivity, excessive alcohol consumption, air pollution. | [Alzheimer’s Society](https://www.alzheimers.org.uk/sites/default/files/pdf/factsheet_risk_factors_for_dementia.pdf); [Livingston2020](https://www.sciencedirect.com/science/article/pii/S0140673620303676?via%3Dihub) |

Note*: Metabolic syndrome includes a combination of raised blood pressure, dyslipidaemia, fatty liver disease, central obesity, and a tendency to develop thrombosis. Note**: medication includes Statins, corticosteroids, and combined treatment with a thiazide diuretic plus a beta-blocker. We did not use medication for identifying post-baseline incident cases in the GP data, due to their lack of certainty for disease ascertainment.


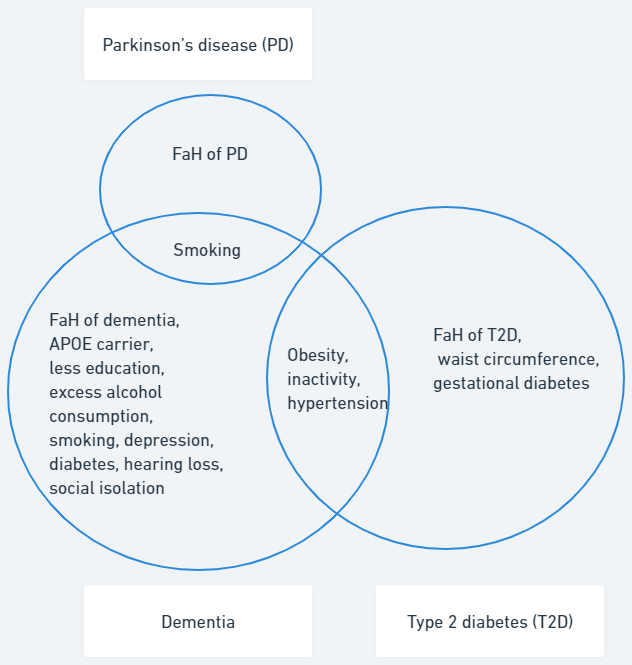


*Supplementary Figure 1. Established/putative risk factors for Parkinson’s Disease (PD), Type 2 diabetes (T2D), and dementia use in this study. FaH: family history. The Venn diagram shows the overlapping risk factors we studied among the three diseases. We note in passing that smoking is a risk factor for dementia, but is inversely associated with PD. Age, sex, and ethnicity are common covariates for all three diseases, and are not shown here.*

Supplementary Table 2. Derivation of variables for 3 conditions of interest. FID: UK Biobank Field ID. FaH: family history. PD: Parkinson’s Disease. T2D: Type 2 diabetes.

| **Variables** | **Description** | **Derivation** |
| --- | --- | --- |
| FaH of PD | No, Yes | Self-reported response on whether father (FID 20107) or mother (FID 20110) or siblings (FID 20111) had PD. |
| Smoking | Current, Previous, Never smokers | Self-reported smoking status (FID 20116). |
| FaH of T2D | No, Yes | Self-reported response on whether father (FID 20107) or mother (FID 20110) or siblings (FID 20111) had diabetes. Note that these fields do not distinguish T2D from diabetes. |
| Waist circumferece | Waist circumference in cm (continuous) | Waist circumference (FID 48) |
| Gestational diabetes | No, Yes | See “Prevalence algorithm 1” in [Eastwood2016](https://www.ncbi.nlm.nih.gov/pmc/articles/PMC5025160/). |
| FaH of dementia | No, Yes | Self-reported response on whether father (FID 20107) or mother (FID 20110) or siblings (FID 20111) had Alzheimer's disease/dementia. Note that these fields do not distinguish dementia from Alzheimer's disease/dementia. |
| APOE $\varepsilon4$ carrier | Not a carrier, $\varepsilon4$ carrier | APOE $\varepsilon4$ status was derived using the APOE SNPs rs429358 and rs7412, which were directly genotyped. |
| Less education | Below GCSE, Equivalent or above GCSE | Self-reported highest qualification (FID 6138 and FID 10722). |
| Excess alcohol consumption | Never, special occasions only, one to three times a month, once or twice a week, three or four times a week, daily or almost daily | Self-reported alcohol intake frequency (FID 1558).  We originally derived excess alcohol consumption using self-reported drinking status (FID 1558); frequency and quantity and types of alcohol consumed per week (FID 1568, FID 1578, FID 1588, FID 1598, FID 1608, FID 5364). However, the missingness of this variable is too high (~43.5%) to be usable. |
| Physical inactivity | No, Yes | Whether a person met the 2017 UK Physical activity guidelines of 150 minutes of moderate activity per week or 75 minutes of vigorous activity (FID 22035). |
| Depression | No, Yes | Self-reported response on whether participants had ever seen their doctor for anxiety or depression (FID 2100 and FID 2090). Positive responses to any of the questions were considered evidence of depression. |
| Diabetes | No, Yes | In T2D analysis, this variable was used as an exclusion critieria and were derived using self-reported fields according to “Prevalence algorithm 1” in [Eastwood2016](https://www.ncbi.nlm.nih.gov/pmc/articles/PMC5025160/) and hospital records at baseline.  In dementia analysis, this variable was used as a covariate and was derived using self-reported diagnosis (FID 2443) or use of diabetes medication (FID 6177, 6153). Positive responses to any of the questions were considered evidence of diabetes. |
| Hearing loss | No, Yes | Self-reported problems with hearing (FID 2247, FID 10793); use of hearing aid (FID 3393). Deaf people (~146 ppl in the whole UKB population) were categorised as having hearing loss. Positive responses to any of the questions were considered evidence of hearing loss. |
| Hypertension | No, Yes | Same derivation approach as [Tapela2020](https://openheart.bmj.com/content/8/1/e001461.abstract). |
| Social isolation | No, Yes | Same derivation approach as [Stevenson2022](https://alz-journals.onlinelibrary.wiley.com/doi/10.1002/alz.12416). |
| Obesity | Underweight/Normal, Overweight, Obese | BMI in Kg/m^2^ (FID 21001) below 18.5 was considered “underweight”, between 18.5 and 25 was “normal”, between 25 and 30 was “overweight” and above 30 was “obese”. |

Missing data: For variables derived using multiple UKB fields (e.g. hearing loss), if all fields used to derive a variable were missing, then the derived variable is regarded as missing.

Supplementary Table 3. The definition of prevalent and incident cases, for the “HES only” and “HES+GP” populations, respectively. HES: hospital inpatient data. Death: death registry.

|  | **“HES only” population** | **“HES+GP” population** |
| --- | --- | --- |
| **Prevalent** | Self-reported diagnosis or medications/HES diagnosis | Self-reported diagnosis or medications/HES diagnosis/GP diagnosis or medications |
| **Incident** | HES diagnosis/Death | HES diagnosis/Death/GP diagnosis |

# Figures and tables

## Study populations


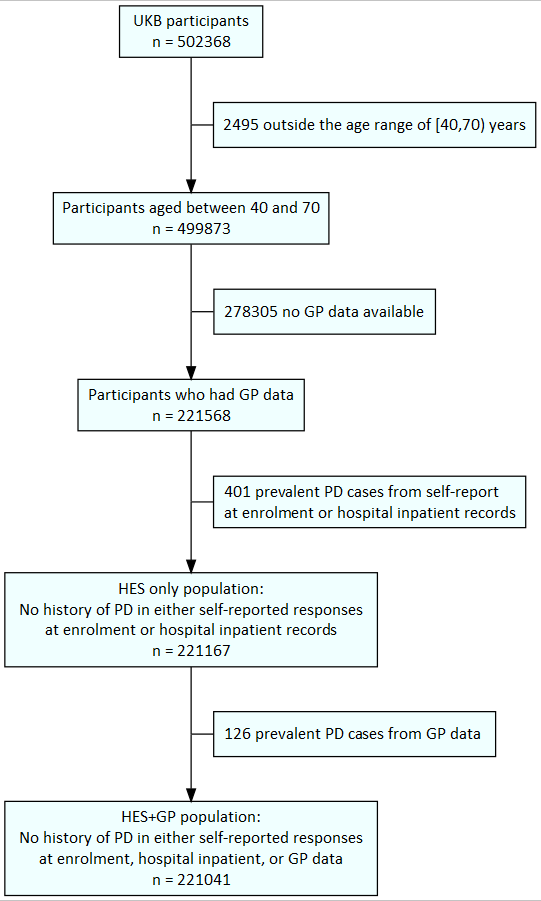


Supplementary Figure 2. Flowchart illustrating “HES only” and “HES+GP” study populations, for Parkinson’s disease (PD).


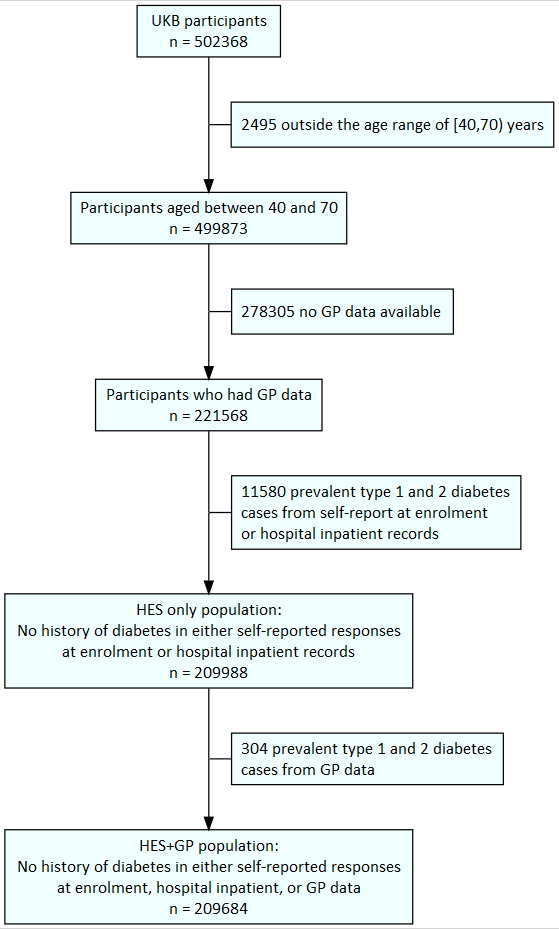


Supplementary Figure 3. Flowchart illustrating “HES only” and “HES+GP” study populations, for type 2 diabetes (T2D).


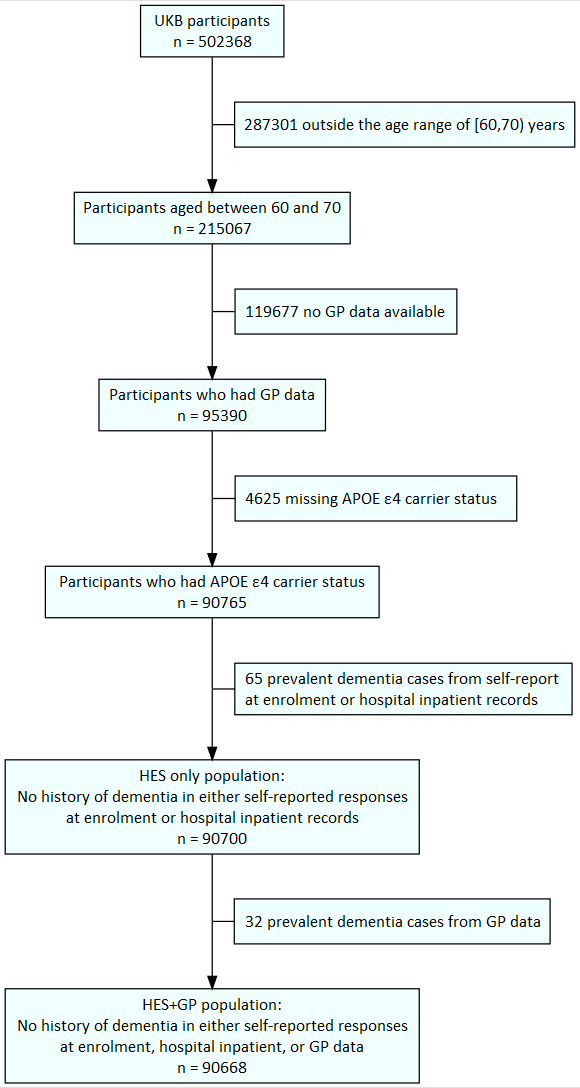


Supplementary Figure 4. Flowchart illustrating “HES only” and “HES+GP” study populations, for dementia. Note that we further excluded people less than 60 years old, yielding a smaller population than for Parkinson’s disease and diabetes.

## Baseline characteristics

Supplementary Table 4. Baseline characteristics of the “HES only” population for Parkinson’s disease (PD), type 2 diabetes (T2D), and dementia. Family history represents family history of Parkinson’s disease, type 2 diabetes, and Alzheimer’s disease/dementia, respectively.

|  | PD (N=221167) | T2D (N=209988) | Dementia (N=90700) |
| --- | --- | --- | --- |

| **Age at enrolment** |  |  |  |
| --- | --- | --- | --- |
| Mean (SD) | 56.99 (8.02) | 56.83 (8.03) | 64.55 (2.81) |
| Min, Max | 40.11, 69.99 | 40.11, 69.99 | 60.00, 69.99 |
| **Self-reported ethnicity** |  |  |  |
| White | 209762 (94.8%) | 199969 (95.2%) | 88165 (97.2%) |
| Black | 2442 (1.1%) | 2190 (1.0%) | 403 (0.4%) |
| S. Asian | 3831 (1.7%) | 3163 (1.5%) | 951 (1.0%) |
| Mixed | 1115 (0.5%) | 1060 (0.5%) | 202 (0.2%) |
| Other | 2965 (1.3%) | 2648 (1.3%) | 609 (0.7%) |
| Missing | 1052 (0.5%) | 958 (0.5%) | 370 (0.4%) |
| **Gender** |  |  |  |
| Female | 121125 (54.8%) | 116882 (55.7%) | 47987 (52.9%) |
| Male | 100042 (45.2%) | 93106 (44.3%) | 42713 (47.1%) |
| **Townsend Deprivation Index** |  |  |  |
| Mean (SD) | -1.32 (3.04) | -1.37 (3.01) | -1.56 (2.92) |
| Min, Max | -6.26, 11.00 | -6.26, 11.00 | -6.26, 10.50 |
| Missing | 324 | 304 | 91 |
| **Family history*** |  |  |  |
| No | 212308 (96.0%) | 167356 (79.7%) | 76956 (84.8%) |
| Yes | 8859 (4.0%) | 42632 (20.3%) | 13744 (15.2%) |
| **Waist circumference (cm)** |  |  |  |
| Mean (SD) |  | 89.63 (13.10) |  |
| Min, Max |  | 20.00, 182.00 |  |
| Missing |  | 827 |  |
| **Gestational diabetes** |  |  |  |
| No |  | 209620 (99.8%) |  |
| Yes |  | 368 (0.2%) |  |
| **ApoE e4 carrier** |  |  |  |
| Not a carrier |  |  | 66785 (73.6%) |
| e4 carrier |  |  | 23915 (26.4%) |
| **Education** |  |  |  |
| Below GCSE |  |  | 26898 (29.7%) |
| Equivalent or above GCSE |  |  | 62508 (68.9%) |
| Missing |  |  | 1294 (1.4%) |
| **Alcohol intake frequency** |  |  |  |
| Never |  |  | 7933 (8.7%) |
| Special occasions only |  |  | 10873 (12.0%) |
| One to three times a month |  |  | 9107 (10.0%) |
| Once or twice a week |  |  | 22083 (24.3%) |
| Three or four times a week |  |  | 19992 (22.0%) |
| Daily or almost daily |  |  | 20566 (22.7%) |
| Missing |  |  | 146 (0.2%) |
| **Physical inactivity** |  |  |  |
| No |  | 92376 (44.0%) | 40540 (44.7%) |
| Yes |  | 76742 (36.5%) | 30627 (33.8%) |
| Missing |  | 40870 (19.5%) | 19533 (21.5%) |
| **Smoking status** |  |  |  |
| Never | 120805 (54.6%) |  | 44908 (49.5%) |
| Previous | 75998 (34.4%) |  | 37851 (41.7%) |
| Current | 23202 (10.5%) |  | 7439 (8.2%) |
| Missing | 1162 (0.5%) |  | 502 (0.6%) |
| **Depression** |  |  |  |
| No |  |  | 60328 (66.5%) |
| Yes |  |  | 30173 (33.3%) |
| Missing |  |  | 199 (0.2%) |
| **Diabetes** |  |  |  |
| No |  |  | 84211 (92.8%) |
| Yes |  |  | 6380 (7.0%) |
| Missing |  |  | 109 (0.1%) |
| **Hearing loss** |  |  |  |
| No |  |  | 61698 (68.0%) |
| Yes |  |  | 28161 (31.0%) |
| Missing |  |  | 841 (0.9%) |
| **Hypertension** |  |  |  |
| No |  | 93935 (44.7%) | 25965 (28.6%) |
| Yes |  | 116053 (55.3%) | 64735 (71.4%) |
| **Social isolation** |  |  |  |
| No |  |  | 82668 (91.1%) |
| Yes |  |  | 7947 (8.8%) |
| Missing |  |  | 85 (0.1%) |
| **BMI** |  |  |  |
| Underweight/Normal |  | 69826 (33.3%) | 26417 (29.1%) |
| Overweight |  | 89861 (42.8%) | 40857 (45.0%) |
| Obese |  | 49082 (23.4%) | 23071 (25.4%) |
| Missing |  | 1219 (0.6%) | 355 (0.4%) |

Supplementary Table 5. Baseline characteristics of the “HES+GP” population, similar to Supplementary Table 4 above.

|  | PD (N=221041) | T2D (N=209684) | Dementia (N=90668) |
| --- | --- | --- | --- |
| **Age at enrolment** |  |  |  |
| Mean (SD) | 56.99 (8.02) | 56.83 (8.03) | 64.55 (2.81) |
| Min, Max | 40.11, 69.99 | 40.11, 69.99 | 60.00, 69.99 |
| **Self-reported ethnicity** |  |  |  |
| White | 209643 (94.8%) | 199722 (95.2%) | 88134 (97.2%) |
| Black | 2441 (1.1%) | 2187 (1.0%) | 403 (0.4%) |
| S. Asian | 3829 (1.7%) | 3140 (1.5%) | 950 (1.0%) |
| Mixed | 1113 (0.5%) | 1056 (0.5%) | 202 (0.2%) |
| Other | 2964 (1.3%) | 2633 (1.3%) | 609 (0.7%) |
| Missing | 1051 (0.5%) | 946 (0.5%) | 370 (0.4%) |
| **Gender** |  |  |  |
| Female | 121043 (54.8%) | 116750 (55.7%) | 47977 (52.9%) |
| Male | 99998 (45.2%) | 92934 (44.3%) | 42691 (47.1%) |
| **Townsend Deprivation Index** |  |  |  |
| Mean (SD) | -1.32 (3.04) | -1.38 (3.01) | -1.56 (2.92) |
| Min, Max | -6.26, 11.00 | -6.26, 11.00 | -6.26, 10.50 |
| Missing | 323 | 304 | 91 |
| **Family history*** |  |  |  |
| No | 212191 (96.0%) | 167165 (79.7%) | 76929 (84.8%) |
| Yes | 8850 (4.0%) | 42519 (20.3%) | 13739 (15.2%) |
| **Waist circumference (cm)** |  |  |  |
| Mean (SD) |  | 89.62 (13.10) |  |
| Min, Max |  | 20.00, 182.00 |  |
| Missing |  | 804 |  |
| **Gestational diabetes** |  |  |  |
| No |  | 209338 (99.8%) |  |
| Yes |  | 346 (0.2%) |  |
| **ApoE e4 carrier** |  |  |  |
| Not a carrier |  |  | 66771 (73.6%) |
| e4 carrier |  |  | 23897 (26.4%) |
| **Education** |  |  |  |
| Below GCSE |  |  | 26883 (29.6%) |
| Equivalent or above GCSE |  |  | 62492 (68.9%) |
| Missing |  |  | 1293 (1.4%) |
| **Alcohol intake frequency** |  |  |  |
| Never |  |  | 7931 (8.7%) |
| Special occasions only |  |  | 10865 (12.0%) |
| One to three times a month |  |  | 9106 (10.0%) |
| Once or twice a week |  |  | 22075 (24.3%) |
| Three or four times a week |  |  | 19987 (22.0%) |
| Daily or almost daily |  |  | 20558 (22.7%) |
| Missing |  |  | 146 (0.2%) |
| **Physical inactivity** |  |  |  |
| No |  | 92272 (44.0%) | 40526 (44.7%) |
| Yes |  | 76621 (36.5%) | 30615 (33.8%) |
| Missing |  | 40791 (19.5%) | 19527 (21.5%) |
| **Smoking status** |  |  |  |
| Never | 120749 (54.6%) |  | 44888 (49.5%) |
| Previous | 75943 (34.4%) |  | 37841 (41.7%) |
| Current | 23187 (10.5%) |  | 7437 (8.2%) |
| Missing | 1162 (0.5%) |  | 502 (0.6%) |
| **Depression** |  |  |  |
| No |  |  | 60312 (66.5%) |
| Yes |  |  | 30157 (33.3%) |
| Missing |  |  | 199 (0.2%) |
| **Diabetes** |  |  |  |
| No |  |  | 84187 (92.9%) |
| Yes |  |  | 6372 (7.0%) |
| Missing |  |  | 109 (0.1%) |
| **Hearing loss** |  |  |  |
| No |  |  | 61687 (68.0%) |
| Yes |  |  | 28140 (31.0%) |
| Missing |  |  | 841 (0.9%) |
| **Hypertension** |  |  |  |
| No |  | 93860 (44.8%) | 25958 (28.6%) |
| Yes |  | 115824 (55.2%) | 64710 (71.4%) |
| **Social isolation** |  |  |  |
| No |  |  | 82641 (91.1%) |
| Yes |  |  | 7942 (8.8%) |
| Missing |  |  | 85 (0.1%) |
| **BMI** |  |  |  |
| Underweight/Normal |  | 69783 (33.3%) | 26405 (29.1%) |
| Overweight |  | 89760 (42.8%) | 40847 (45.1%) |
| Obese |  | 48946 (23.3%) | 23061 (25.4%) |
| Missing |  | 1195 (0.6%) | 355 (0.4%) |

## Venn diagrams

The detailed Venn diagrams here provide additional information on incident cases from the combined HES and death registry (i.e. HES/Dth) and GP records of PD, T2D, and dementia, respectively. For people with both GP and HES diagnoses during the full follow-up (i.e. until the HES/Dth censoring date) the histograms show the time difference between these diagnoses.


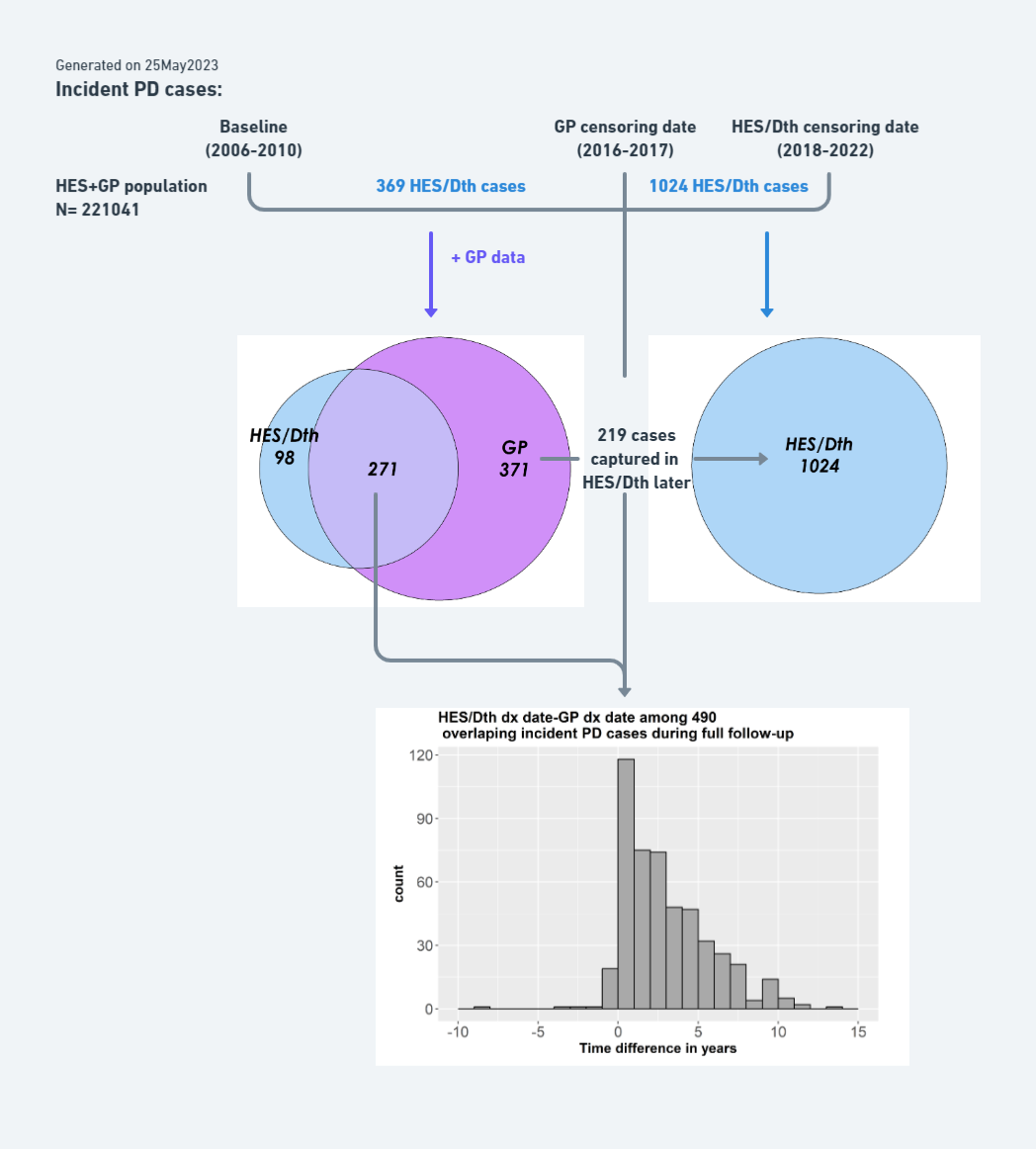


Supplementary Figure 5. Venn diagram comparing incident cases of Parkinson’s Disease (PD) from HES/Dth and those from GP records. Among the 371 cases in GP (but not in HES/Dth) data prior to GP censoring date, 219 would appear in HES/Dth later; this means that we would obtain 152 (= 371 - 219) new cases from GP data whether we censor GP data or not. If we do not censor GP data, we would further gain 805 (= 1024 - 219) PD cases. For the 490 participants whose disease diagnoses are present in both HES and GP data during full follow-up, the histogram shows the time difference (i.e. lag) of the diagnosis dates between these two data sources; the median of time difference is 2.31 years, and the mean is 2.99 (SD=2.74) years.


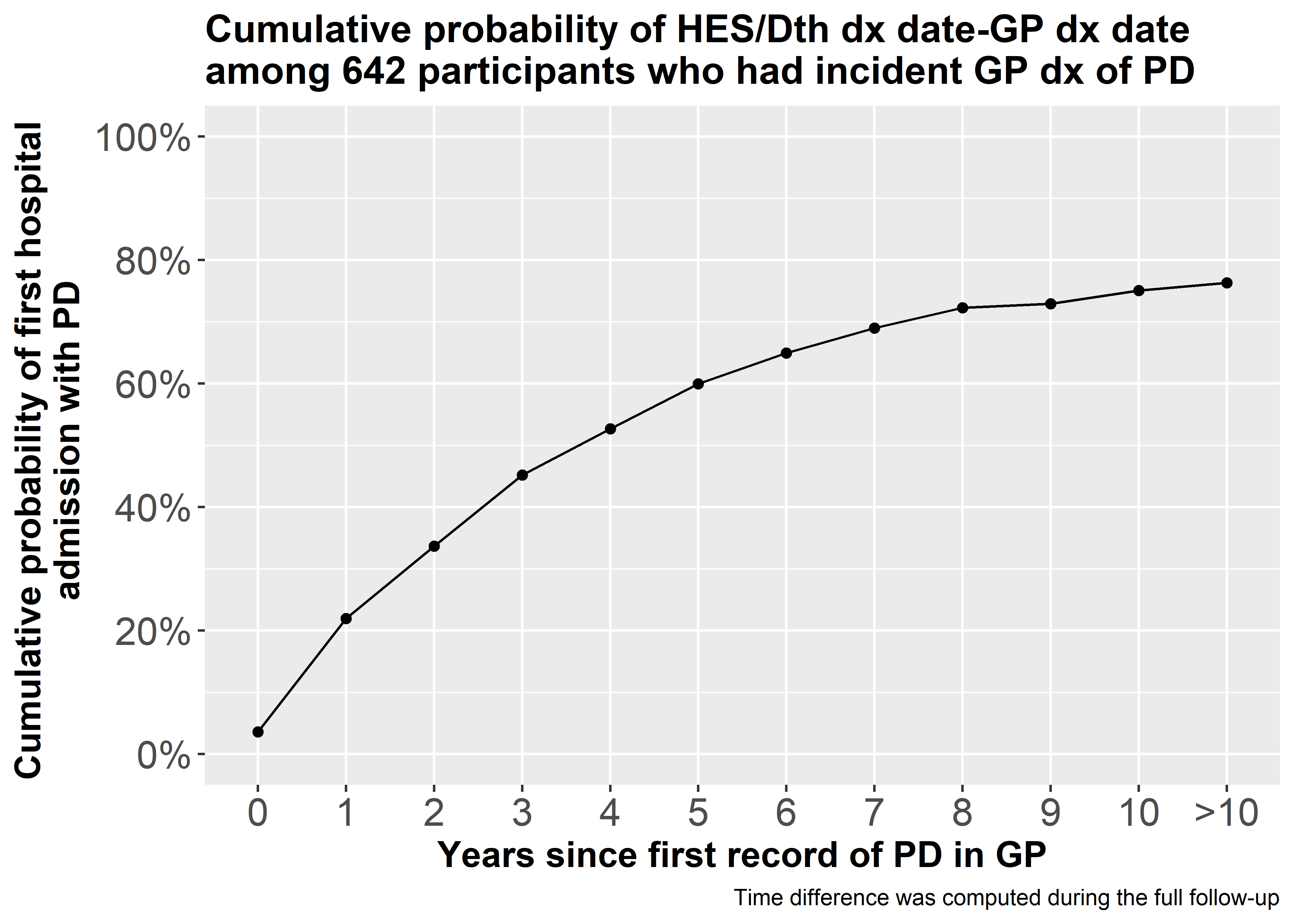


Supplementary Figure 6. Cumulative probability of first admission to hospital with Parkinson’s disease (PD) or death caused by PD (referred to as “HES diagnosis”) since first diagnosis of PD in primary care (referred to as “GP diagnosis”) among the 642 participants who had incident GP diagnosis of PD. Each point shows $\frac{\#(HES diag-GP diag \leq year)}{\# GP diag}$ computed during full follow-up, where “diag” stands for “diagnosis”, and “#” means “the number of”. Note that the cumulative probability plot does not start from 0% because some people had HES diagnosis of PD before GP diagnosis.


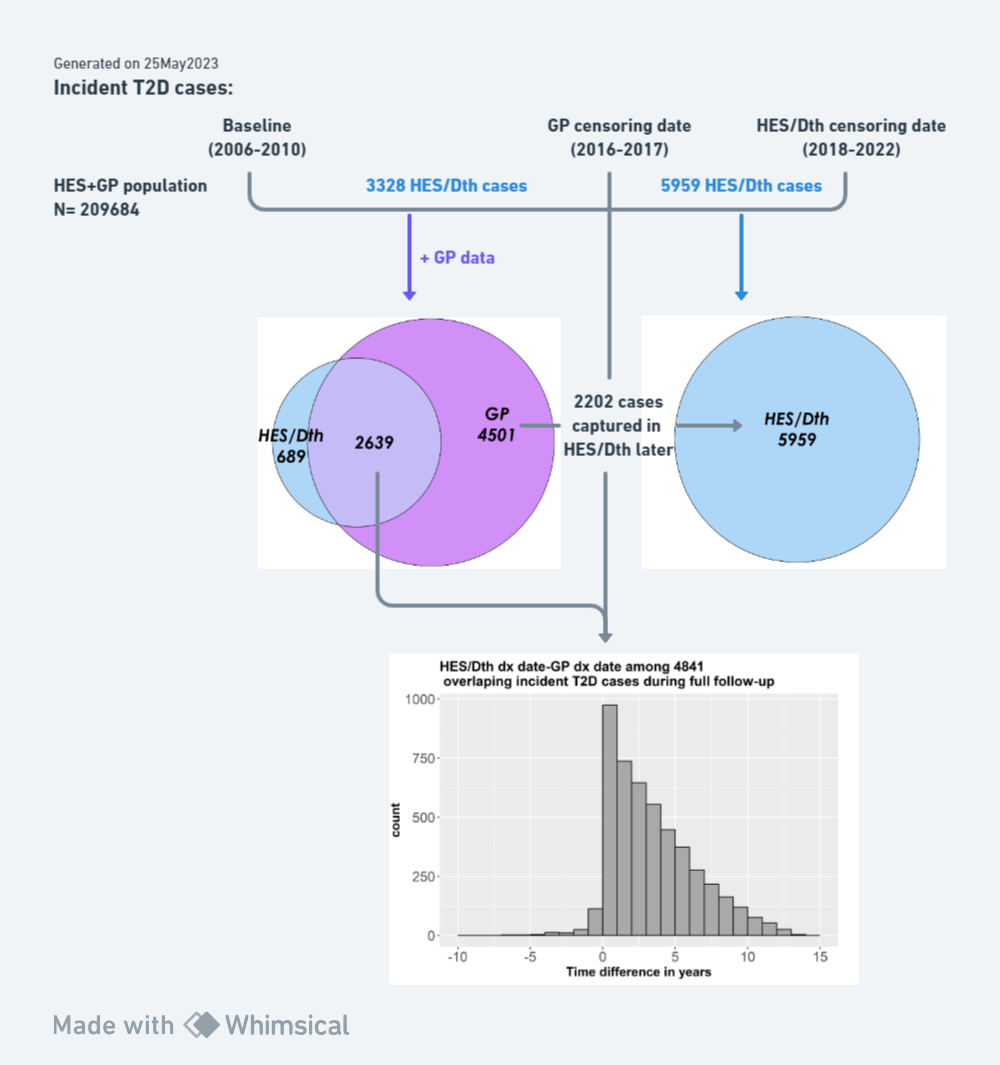


Supplementary Figure 7. Venn diagram comparing incident cases of type 2 diabetes (T2D) from HES/Dth and those from GP records. Among the 4501 cases in GP (but not in HES/Dth) data prior to GP censoring date, 2202 would appear in HES/Dth later; this means that we would obtain 2299 (= 4501 - 2202) new cases from GP data whether we censor GP data or not. If we do not censor GP data, we would further gain 3757 (= 5959 - 2202) T2D cases. For the 4841 participants whose disease diagnoses are present in both HES and GP data during full follow-up, the histogram shows the time difference (i.e. lag) of the diagnosis dates between these two data sources; the median of time difference is 2.82 years, and the mean is 3.45 (SD=2.96) years.


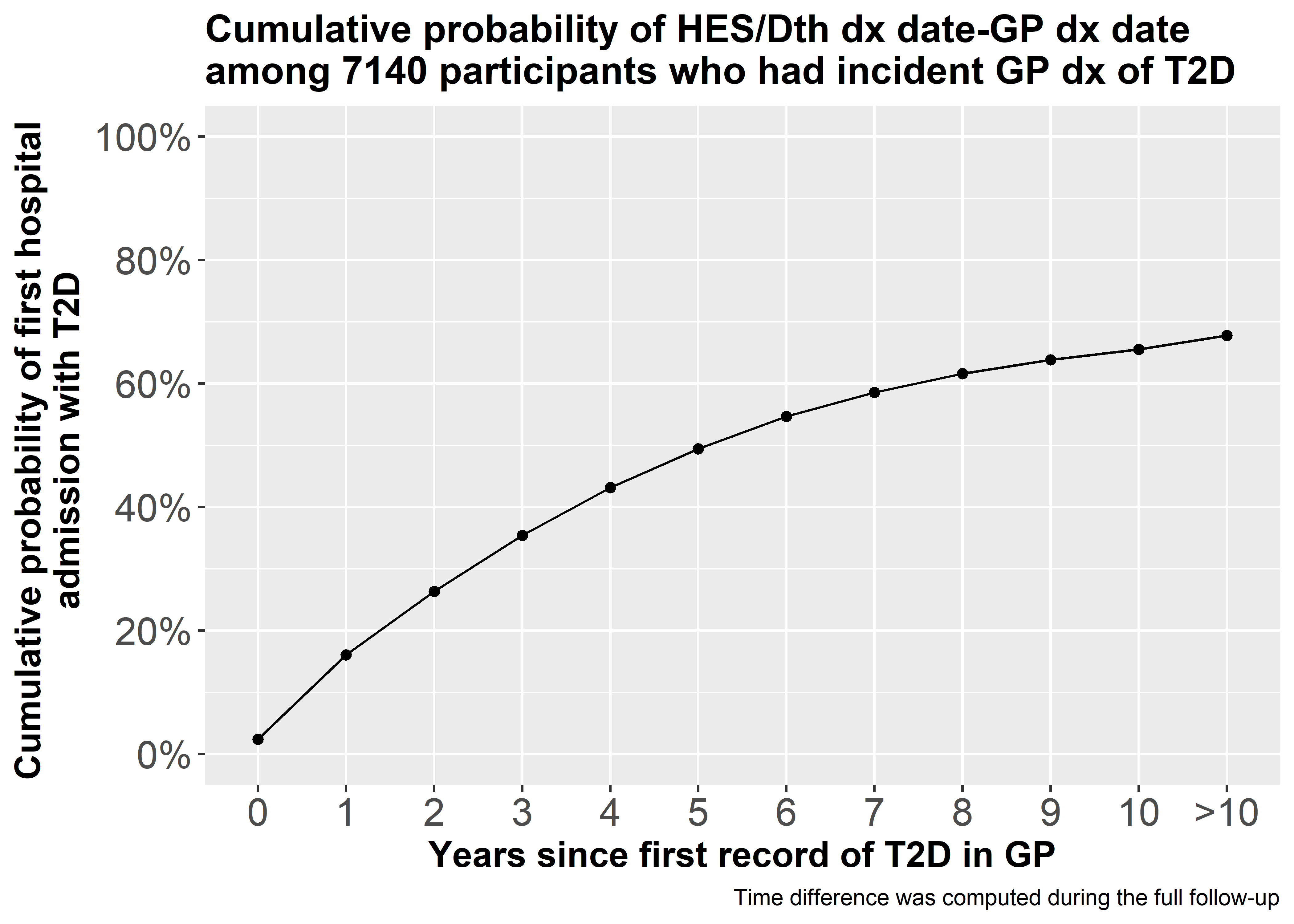


Supplementary Figure 8. Cumulative probability of first admission to hospital with type 2 diabetes (T2D) or death caused by T2D since first diagnosis of T2D in primary care, among the 7140 participants who had incident GP diagnosis of T2D.


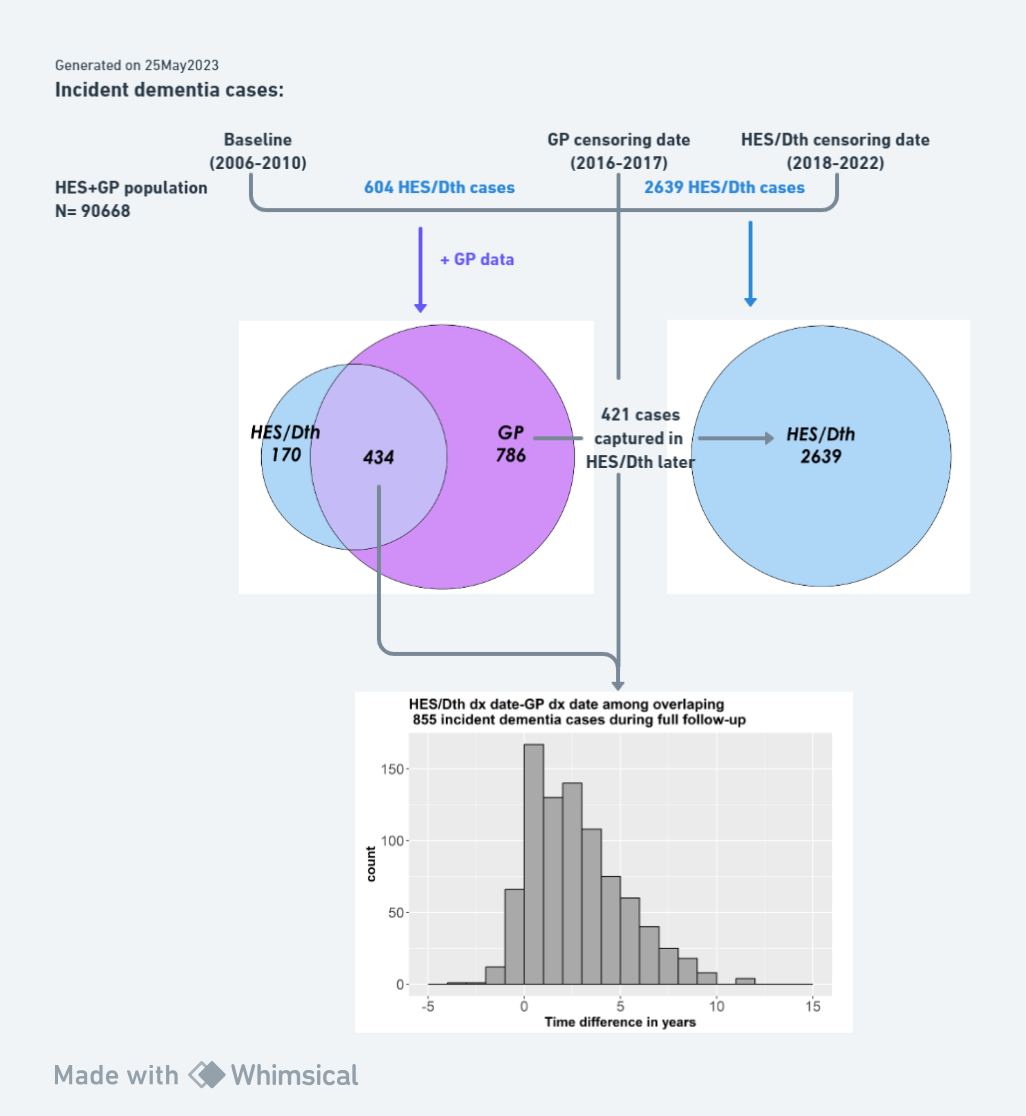


Supplementary Figure 9. Venn diagram comparing incident cases of dementia from HES/Dth and those from GP records. Among the 786 cases in GP (but not in HES/Dth) data prior to GP censoring date, 421 would appear in HES/Dth later; this means that we would obtain 365 (= 786 - 421) new cases from GP data whether we censor GP data or not. If we do not censor GP data, we would further gain 2218 (= 2639 - 421) dementia cases. For the 855 participants whose disease diagnoses are present in both HES and GP data during full follow-up, the histogram shows the time difference (i.e. lag) of the diagnosis dates between these two data sources; the median of time difference is 2.25 years, and the mean is 2.73 (SD=2.44) years.


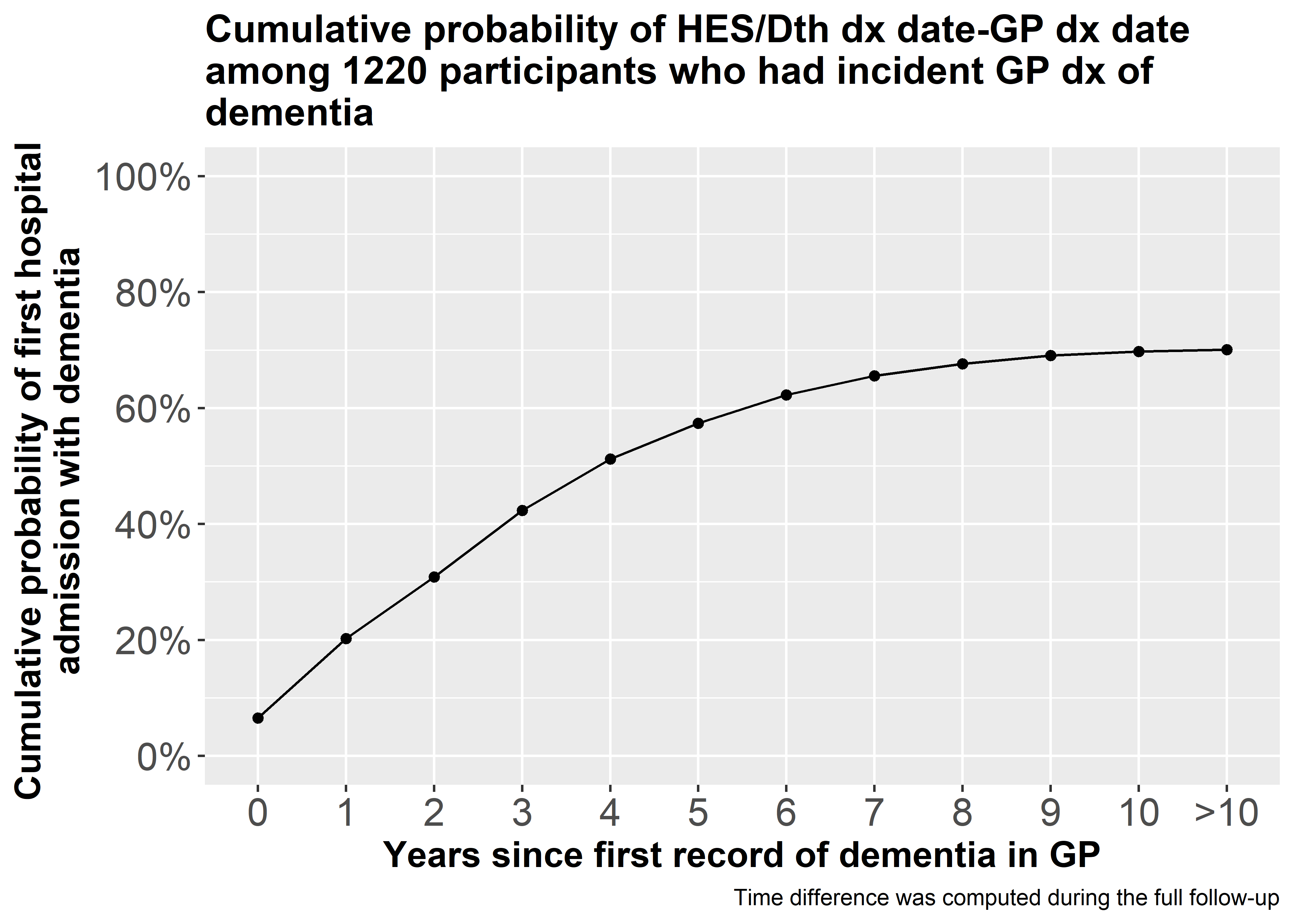


Supplementary Figure 10. Cumulative probability of first admission to hospital with dementia or death caused by dementia since the first diagnosis of dementia in primary care, among the 1220 participants who had incident GP diagnosis of dementia.

# Further discussion

We note some studies used separate follow-up periods when comparing across different data sources, resulting in longer follow-up periods than those in this paper, akin to the alternative censoring approach discussed below. Consequently, the effect estimates from our models are likely to be different from those in the existing literature, albeit largely in the same direction.

We demonstrated that it is beneficial to include GP data in the UKB cohort, for the three chosen diseases that are primarily managed in primary care. However, for diseases that are likely to be presented in hospital prior to GP record (e.g. myocardial infarction), the added benefit of GP data may be less prominent than that in this study.

We excluded people with a GP diagnosis but without a HES diagnosis when computing the summary statistics of the time difference. Possible reasons for not having a HES diagnosis include death from other causes, not being admitted to hospital within the follow-up period, not having the disease recorded during an admission, or the follow-up period being short as described in the main paper.

## Alternative study population

In this project, we restricted our analysis to the 45% UKB participants whose GP data are available. An alternative study population can be all UKB participants, which is the natural analytical choice in the absence of GP data, reflecting the “HES only” analysis in practice. In this instance, the corresponding “HES+GP” analysis answers the question “what is the added value of GP data when supplementing HES data with GP data wherever possible?” The obvious drawback is that this is not equal comparison between people with and without GP data, akin to the drawbacks of not censoring GP data (Supplementary Table S.2). This can be a future direction that extends our work in this paper.

## Alternative research question

An alternative research question can be “What is the additional benefit of HES data to the existing GP data?” This question is perhaps best answered by primary care cohorts that is linked to hospital data, such that GP data are plentiful and abundant. However, one could still use the ~45% UKB cohort whose GP data are available. The corresponding study populations would be “GP only” v.s. “GP+HES”.

# An alternative censoring approach

## Administrative censoring dates

The UKB [Showcase](https://biobank.ndph.ox.ac.uk/showcase/exinfo.cgi?src=Data_providers_and_dates) provides the administrative censoring dates of all data sources, including death, hospital inpatient (referred to as HES hereafter), and primary care (GP) data. The administrative censoring dates of GP data are earlier than those of HES/Death data, illustrated in Supplementary Figure 11 below. In this study, we have chosen to censor by GP data (i.e. censor at GP administrative censoring dates), and therefore we did not incorporate all the incident cases that are available in the HES/Death data.

The administrative censoring date of HES/Death data (2018-2022), depending on whether the data came from England, Wales or Scotland, hereafter referred to as the “HES censoring date”) was later than the GP censoring date (2016-2017), as illustrated in Supplementary Figure 11. Therefore, our “HES only” population has fewer events and shorter follow-up periods than what researchers would typically use (i.e. the maximal follow-up period available for HES at the absence of GP data).


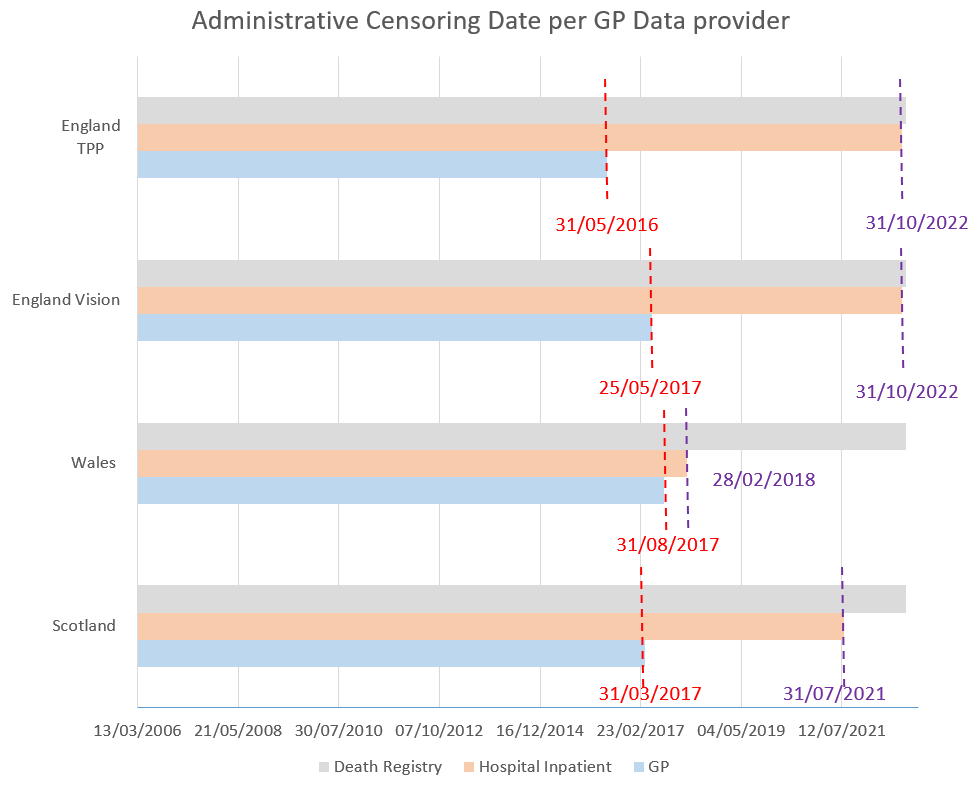


Supplementary Figure 11. Administrative censoring dates for GP, hospital inpatient, and death registry records. Dates in red show the administrative censoring dates for GP, and those in purple show the earlier admin censoring date of hospital inpatient and death registry records (i.e. HES/Dth). We used the record-level hospital inpatient data and death registry, which are updated more frequently than those on the UKB Showcase.

## An alternative censoring approach

In this paper, we have chosen to censor both the “HES only” and “HES+GP” populations by the GP administrative censoring date 2016-2017 (i.e. the “To censor” option in Table 6 below) for assessing the added benefit of GP data, albeit at the cost of fewer incident cases than the “No to censor” option described below. The “To censor” option also allows one to examine the added number of cases captured by HES after the end of GP follow-up (i.e. until the GP censoring date), when comparing “HES only” with “HES+GP” populations.

An alternative censoring approach is “Not to censor” the GP data, and instead use the HES administrative censoring date 2018-2022 (i.e. the “No to censor” option in Table 6 below). This alternative “No to censor” approach yields longer follow-up time and hence providing more incident cases, in contrast with the “To censor” approach in the main analysis of this paper.

In the “Not to censor” approach, participants are censored on the earlier of the following dates for both the “HES only” and “HES+GP” populations:

- Death from causes other than the outcome disease
- Loss to follow-up
- Administrative censoring date (of HES data)

The admin censoring date of HES (not GP) data is used, yielding the following analysis populations that are different from those in the main analysis of this paper:

- “HES only” population: it is the natural choice to censor by the admin censoring date of HES (not GP) data, when researchers do not incorporate GP data in their analysis. Therefore, it closely reflects the research reality.
- “HES+GP” population: By not censoring by GP data, we implicitly regard HES as the primary data source, and GP data as the extra data for supplementing HES.

Supplementary Table 6. Comparing the two options of censoring GP data (to censor or not to censor), each with its pros and cons.

|  | Interpretation | Pros | Cons | Example |
| --- | --- | --- | --- | --- |
| To censor | - Regarding HES and GP as parallel data sources | - Equal treatment to HES and GP data  - Unbiased follow-up | - Shorter follow-up time (HES censored at 2021 vs. GP at 2016)  - Fewer incident cases | This paper |
| Not to censor | - Supplementing HES with GP data (regarding HES as the primary data source) | - Longer follow-up time (HES censored at 2021 vs. GP at 2016)  - More incident cases | - Not treating GP data the same way as HES data  - Potentially biased towards longer follow-up. If a person’s outcome at 2018 in GP and at 2020 in HES, then his outcome will only be captured at 2020 in HES, whereas the actual outcome is at 2018 in GP. This is not a fair comparison for people whose outcomes are captured before 2016 in GP data. | [Bragg2022](https://bmcmedicine.biomedcentral.com/articles/10.1186/s12916-022-02354-9) |

These two different censoring options have impact on the study results. The “To censor” option yields an HR (= 0.96, 95%CI 0.81, 1.14) of “hearing loss” that is in the opposite direction to existing literature, as shown in Figure 7 of the main paper. In contrast, the “No to censor” option produces an HR (> 1) that is consistent with the literature (Supplementary Tables 7-8).

Supplementary Table 7. Multiple imputation results of Cox models of dementia obtained using “HES Only” population with full follow-up (N=90700).

| Coefficient | HR | 95% CI | p |
| --- | --- | --- | --- |
| **Hearing loss** |  |  |  |
| No | 1 |  |  |
| Yes | 1.04 | (0.97, 1.12) | 0.266 |
| **Age at enrolment** | 1.22 | (1.21, 1.24) | <0.001 |
| **Ethnicity** |  |  |  |
| White | 1 |  |  |
| Other ethnicity | 0.95 | (0.77, 1.18) | 0.653 |
| **Gender** |  |  |  |
| Female | 1 |  |  |
| Male | 1.28 | (1.19, 1.38) | <0.001 |
| **Townsend Deprivation Index** | 1.04 | (1.02, 1.05) | <0.001 |
| **Family history of Alzheimer’s disease/dementia** |  |  |  |
| No family history of dem | 1 |  |  |
| Family history of dem | 1.48 | (1.36, 1.61) | <0.001 |
| **ApoE e4 carrier** |  |  |  |
| Not a carrier | 1 |  |  |
| e4 carrier | 3.27 | (3.05, 3.51) | <0.001 |
| **Education** |  |  |  |
| Below GCSE | 1 |  |  |
| Equivalent or above GCSE | 0.75 | (0.70, 0.81) | <0.001 |
| **Alcohol intake frequency** |  |  |  |
| Never | 1 |  |  |
| Special occasions only | 0.79 | (0.70, 0.91) | <0.001 |
| One to three times a month | 0.66 | (0.57, 0.77) | <0.001 |
| Once or twice a week | 0.69 | (0.61, 0.77) | <0.001 |
| Three or four times a week | 0.64 | (0.56, 0.73) | <0.001 |
| Daily or almost daily | 0.63 | (0.56, 0.72) | <0.001 |
| **Physical inactivity** |  |  |  |
| No | 1 |  |  |
| Yes | 1.13 | (1.04, 1.22) | 0.004 |
| **Smoking status** |  |  |  |
| Never | 1 |  |  |
| Previous | 1.14 | (1.05, 1.23) | <0.001 |
| Current | 1.25 | (1.10, 1.42) | <0.001 |
| **Depression** |  |  |  |
| No | 1 |  |  |
| Yes | 1.25 | (1.16, 1.35) | <0.001 |
| **Diabetes** |  |  |  |
| No | 1 |  |  |
| Yes | 1.92 | (1.73, 2.13) | <0.001 |
| **Hypertension** |  |  |  |
| No | 1 |  |  |
| Yes | 1.25 | (1.14, 1.36) | <0.001 |
| **Social isolation** |  |  |  |
| No | 1 |  |  |
| Yes | 1.22 | (1.09, 1.36) | <0.001 |
| **BMI** |  |  |  |
| Underweight/Normal | 1 |  |  |
| Overweight | 0.88 | (0.81, 0.96) | 0.003 |
| Obese | 0.83 | (0.75, 0.92) | <0.001 |

Supplementary Table 8. Complete case results of Cox models of dementia obtained using “HES Only” population with full follow-up (N=69202).

| Coefficient | HR | 95% CI | p |
| --- | --- | --- | --- |
| **Hearing loss** |  |  |  |
| No | 1 |  |  |
| Yes | 1.05 | (0.96, 1.14) | 0.309 |
| **Age at enrolment** | 1.22 | (1.20, 1.24) | <0.001 |
| **Ethnicity** |  |  |  |
| White | 1 |  |  |
| Other ethnicity | 1.03 | (0.79, 1.33) | 0.851 |
| **Gender** |  |  |  |
| Female | 1 |  |  |
| Male | 1.35 | (1.24, 1.47) | <0.001 |
| **Townsend Deprivation Index** | 1.04 | (1.02, 1.05) | <0.001 |
| **Family history of Alzheimer’s disease/dementia** |  |  |  |
| No family history of dem | 1 |  |  |
| Family history of dem | 1.50 | (1.36, 1.66) | <0.001 |
| **ApoE e4 carrier** |  |  |  |
| Not a carrier | 1 |  |  |
| e4 carrier | 3.24 | (2.98, 3.51) | <0.001 |
| **Education** |  |  |  |
| Below GCSE | 1 |  |  |
| Equivalent or above GCSE | 0.73 | (0.67, 0.79) | <0.001 |
| **Alcohol intake frequency** |  |  |  |
| Never | 1 |  |  |
| Special occasions only | 0.85 | (0.72, 1.00) | 0.044 |
| One to three times a month | 0.68 | (0.57, 0.81) | <0.001 |
| Once or twice a week | 0.70 | (0.60, 0.81) | <0.001 |
| Three or four times a week | 0.66 | (0.57, 0.77) | <0.001 |
| Daily or almost daily | 0.65 | (0.56, 0.76) | <0.001 |
| **Physical inactivity** |  |  |  |
| No | 1 |  |  |
| Yes | 1.12 | (1.03, 1.22) | 0.006 |
| **Smoking status** |  |  |  |
| Never | 1 |  |  |
| Previous | 1.17 | (1.07, 1.28) | <0.001 |
| Current | 1.34 | (1.16, 1.56) | <0.001 |
| **Depression** |  |  |  |
| No | 1 |  |  |
| Yes | 1.22 | (1.12, 1.33) | <0.001 |
| **Diabetes** |  |  |  |
| No | 1 |  |  |
| Yes | 1.79 | (1.58, 2.04) | <0.001 |
| **Hypertension** |  |  |  |
| No | 1 |  |  |
| Yes | 1.21 | (1.10, 1.34) | <0.001 |
| **Social isolation** |  |  |  |
| No | 1 |  |  |
| Yes | 1.27 | (1.12, 1.44) | <0.001 |
| **BMI** |  |  |  |
| Underweight/Normal | 1 |  |  |
| Overweight | 0.85 | (0.77, 0.93) | <0.001 |
| Obese | 0.80 | (0.72, 0.90) | <0.001 |

# Outputs of Cox models (multiple imputation)

Supplementary Table 9. Multiple imputation results of Cox models of PD obtained using HES only (N=221167) and HES+GP population (N=221041).

|  | HES only |  | HES+GP |  |
| --- | --- | --- | --- | --- |
| Coefficient | HR (95% CI) | p | HR (95% CI) | p |
| Age at enrolment | 1.16 (1.13, 1.18) | <0.001 | 1.13 (1.11, 1.14) | <0.001 |
| Ethnicity |  |  |  |  |
| White | 1 |  | 1 |  |
| Other ethnicity | 0.88 (0.45, 1.71) | 0.701 | 0.96 (0.62, 1.48) | 0.844 |
| Gender |  |  |  |  |
| Female | 1 |  | 1 |  |
| Male | 1.64 (1.33, 2.02) | <0.001 | 1.91 (1.65, 2.22) | <0.001 |
| Townsend Deprivation Index | 1.02 (0.99, 1.06) | 0.191 | 1.01 (0.98, 1.03) | 0.509 |
| Family history of PD |  |  |  |  |
| No family history of PD | 1 |  | 1 |  |
| Family history of PD | 1.86 (1.29, 2.69) | <0.001 | 2.10 (1.63, 2.70) | <0.001 |
| Smoking status |  |  |  |  |
| Never | 1 |  | 1 |  |
| Previous | 0.99 (0.80, 1.22) | 0.915 | 0.86 (0.74, 1.00) | 0.056 |
| Current | 0.62 (0.40, 0.97) | 0.038 | 0.49 (0.35, 0.69) | <0.001 |

Supplementary Table 10. Multiple imputation results of Cox models of T2D obtained using male subgroup of HES only (N=93106) and male subgroup of HES+GP population (N=92934).

|  | HES only |  | HES+GP |  |
| --- | --- | --- | --- | --- |
| Coefficient | HR (95% CI) | p | HR (95% CI) | p |
| Age at enrolment | 1.05 (1.05, 1.06) | <0.001 | 1.03 (1.03, 1.04) | <0.001 |
| Ethnicity |  |  |  |  |
| White | 1 |  | 1 |  |
| Other ethnicity | 2.30 (1.93, 2.74) | <0.001 | 2.80 (2.52, 3.11) | <0.001 |
| Townsend Deprivation Index | 1.07 (1.05, 1.08) | <0.001 | 1.05 (1.04, 1.06) | <0.001 |
| Family history of diabetes |  |  |  |  |
| No family history of Diab | 1 |  | 1 |  |
| Family history of Diab | 1.85 (1.68, 2.04) | <0.001 | 1.81 (1.70, 1.93) | <0.001 |
| Waist circumference (cm) | 1.04 (1.04, 1.05) | <0.001 | 1.04 (1.04, 1.04) | <0.001 |
| BMI |  |  |  |  |
| Underweight/Normal | 1 |  | 1 |  |
| Overweight | 1.45 (1.21, 1.74) | <0.001 | 1.72 (1.51, 1.94) | <0.001 |
| Obese | 2.18 (1.76, 2.69) | <0.001 | 2.85 (2.47, 3.29) | <0.001 |
| Physical inactivity |  |  |  |  |
| No | 1 |  | 1 |  |
| Yes | 1.20 (1.08, 1.32) | <0.001 | 1.27 (1.19, 1.36) | <0.001 |
| Hypertension |  |  |  |  |
| No | 1 |  | 1 |  |
| Yes | 1.70 (1.50, 1.92) | <0.001 | 1.74 (1.61, 1.89) | <0.001 |

Supplementary Table 11. Multiple imputation results of Cox models of T2D obtained using female subgroup of HES only (N=116882) and female subgroup of HES+GP population (N=116750).

|  | HES only |  | HES+GP |  |
| --- | --- | --- | --- | --- |
| Coefficient | HR (95% CI) | p | HR (95% CI) | p |
| Age at enrolment | 1.06 (1.05, 1.06) | <0.001 | 1.04 (1.04, 1.05) | <0.001 |
| Ethnicity |  |  |  |  |
| White | 1 |  | 1 |  |
| Other ethnicity | 2.62 (2.20, 3.12) | <0.001 | 2.81 (2.51, 3.14) | <0.001 |
| Townsend Deprivation Index | 1.05 (1.03, 1.07) | <0.001 | 1.04 (1.03, 1.05) | <0.001 |
| Family history of diabetes |  |  |  |  |
| No family history of Diab | 1 |  | 1 |  |
| Family history of Diab | 1.90 (1.71, 2.12) | <0.001 | 2.00 (1.86, 2.15) | <0.001 |
| Waist circumference (cm) | 1.06 (1.05, 1.06) | <0.001 | 1.05 (1.05, 1.05) | <0.001 |
| BMI |  |  |  |  |
| Underweight/Normal | 1 |  | 1 |  |
| Overweight | 1.60 (1.31, 1.97) | <0.001 | 1.67 (1.46, 1.90) | <0.001 |
| Obese | 1.90 (1.50, 2.39) | <0.001 | 2.19 (1.88, 2.55) | <0.001 |
| Physical inactivity |  |  |  |  |
| No | 1 |  | 1 |  |
| Yes | 1.21 (1.06, 1.38) | 0.006 | 1.14 (1.05, 1.23) | 0.002 |
| Hypertension |  |  |  |  |
| No | 1 |  | 1 |  |
| Yes | 1.74 (1.53, 1.98) | <0.001 | 1.76 (1.62, 1.92) | <0.001 |
| Gestational diabetes |  |  |  |  |
| No | 1 |  | 1 |  |
| Yes | 4.82 (3.28, 7.08) | <0.001 | 3.27 (2.40, 4.46) | <0.001 |

Supplementary Table 12. Multiple imputation results of Cox models of dementia obtained using HES only (N=90700) and HES+GP population (N=90668).

|  | HES only |  | HES+GP |  |
| --- | --- | --- | --- | --- |
| Coefficient | HR (95% CI) | p | HR (95% CI) | p |
| Age at enrolment | 1.24 (1.21, 1.28) | <0.001 | 1.19 (1.16, 1.21) | <0.001 |
| Ethnicity |  |  |  |  |
| White | 1 |  | 1 |  |
| Other ethnicity | 0.86 (0.52, 1.43) | 0.565 | 1.18 (0.87, 1.61) | 0.280 |
| Gender |  |  |  |  |
| Female | 1 |  | 1 |  |
| Male | 1.52 (1.28, 1.80) | <0.001 | 1.31 (1.17, 1.47) | <0.001 |
| Townsend Deprivation Index | 1.04 (1.01, 1.07) | 0.003 | 1.04 (1.02, 1.06) | <0.001 |
| Family history of Alzheimer’s disease/dementia |  |  |  |  |
| No family history of dem | 1 |  | 1 |  |
| Family history of dem | 1.68 (1.39, 2.02) | <0.001 | 1.76 (1.56, 1.99) | <0.001 |
| ApoE e4 carrier |  |  |  |  |
| Not a carrier | 1 |  | 1 |  |
| e4 carrier | 3.20 (2.73, 3.75) | <0.001 | 3.12 (2.81, 3.47) | <0.001 |
| Education |  |  |  |  |
| Below GCSE | 1 |  | 1 |  |
| Equivalent or above GCSE | 0.84 (0.71, 1.00) | 0.057 | 0.76 (0.68, 0.85) | <0.001 |
| Alcohol intake frequency |  |  |  |  |
| Never | 1 |  | 1 |  |
| Special occasions only | 0.77 (0.59, 1.02) | 0.066 | 0.77 (0.63, 0.94) | 0.010 |
| One to three times a month | 0.40 (0.28, 0.58) | <0.001 | 0.66 (0.52, 0.82) | <0.001 |
| Once or twice a week | 0.59 (0.46, 0.77) | <0.001 | 0.69 (0.57, 0.82) | <0.001 |
| Three or four times a week | 0.51 (0.38, 0.68) | <0.001 | 0.63 (0.52, 0.77) | <0.001 |
| Daily or almost daily | 0.53 (0.40, 0.70) | <0.001 | 0.62 (0.51, 0.75) | <0.001 |
| Physical inactivity |  |  |  |  |
| No | 1 |  | 1 |  |
| Yes | 1.08 (0.89, 1.30) | 0.440 | 1.07 (0.93, 1.22) | 0.361 |
| Smoking status |  |  |  |  |
| Never | 1 |  | 1 |  |
| Previous | 1.00 (0.84, 1.19) | 0.971 | 0.93 (0.83, 1.04) | 0.202 |
| Current | 1.20 (0.91, 1.58) | 0.187 | 1.01 (0.83, 1.23) | 0.922 |
| Depression |  |  |  |  |
| No | 1 |  | 1 |  |
| Yes | 1.39 (1.18, 1.64) | <0.001 | 1.18 (1.05, 1.32) | 0.004 |
| Diabetes |  |  |  |  |
| No | 1 |  | 1 |  |
| Yes | 2.13 (1.70, 2.68) | <0.001 | 1.99 (1.70, 2.34) | <0.001 |
| Hearing loss |  |  |  |  |
| No | 1 |  | 1 |  |
| Yes | 0.96 (0.81, 1.14) | 0.676 | 1.05 (0.94, 1.18) | 0.383 |
| Hypertension |  |  |  |  |
| No | 1 |  | 1 |  |
| Yes | 1.10 (0.90, 1.34) | 0.351 | 1.07 (0.94, 1.21) | 0.318 |
| Social isolation |  |  |  |  |
| No | 1 |  | 1 |  |
| Yes | 1.55 (1.24, 1.95) | <0.001 | 1.20 (1.01, 1.42) | 0.035 |
| BMI |  |  |  |  |
| Underweight/Normal | 1 |  | 1 |  |
| Overweight | 0.81 (0.66, 0.98) | 0.030 | 0.84 (0.74, 0.96) | 0.009 |
| Obese | 0.81 (0.65, 1.02) | 0.073 | 0.77 (0.66, 0.89) | <0.001 |

# Outputs of Cox models (complete case)


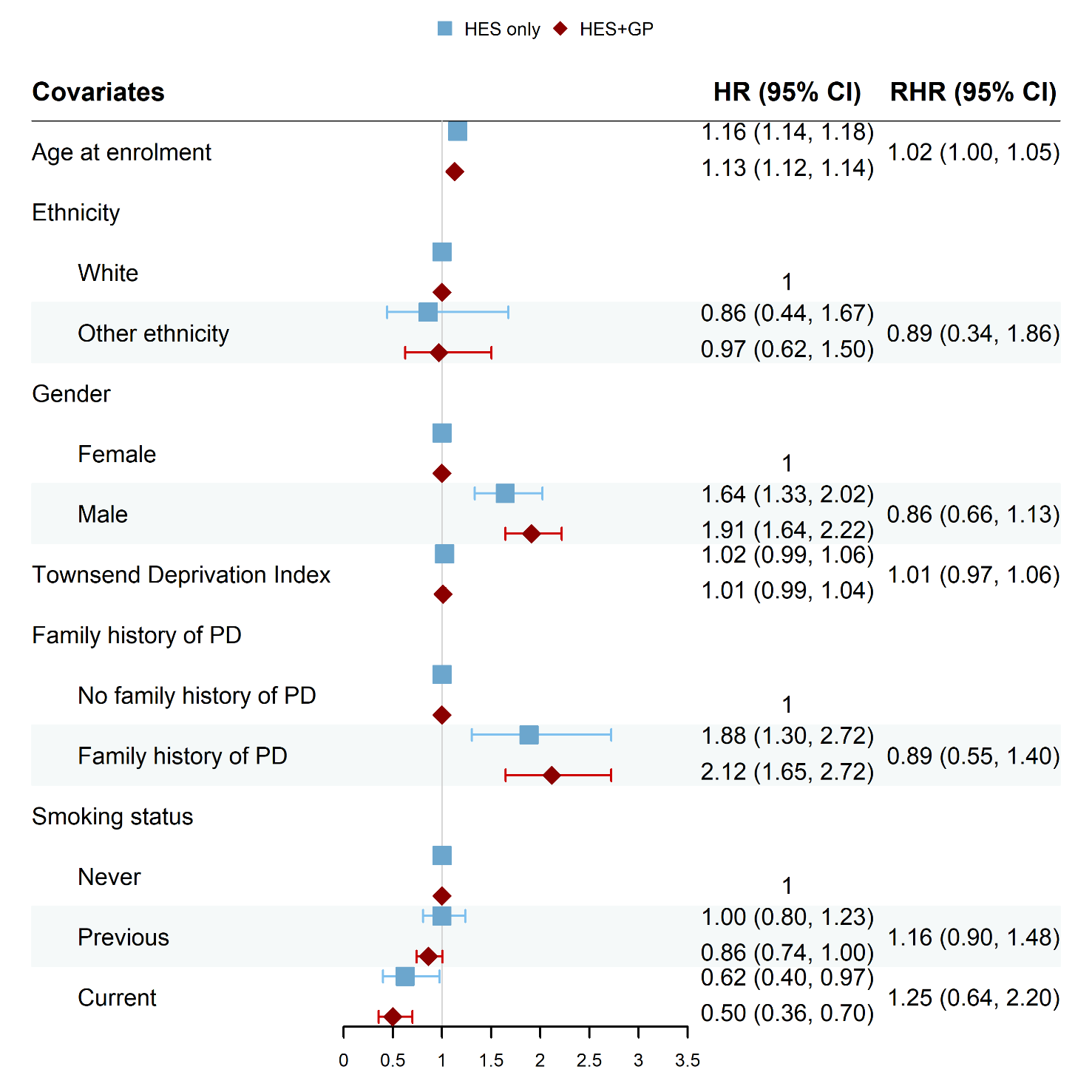


Supplementary Figure 12. Forest plot for Parkinson's disease (complete case)

Supplementary Table 13. Complete case results of Cox models of PD obtained using HES only (N=218992) and HES+GP population (N=218868).

|  | HES only |  | HES+GP |  |
| --- | --- | --- | --- | --- |
| Coefficient | HR (95% CI) | p | HR (95% CI) | p |
| Age at enrolment | 1.16 (1.14, 1.18) | <0.001 | 1.13 (1.12, 1.14) | <0.001 |
| Ethnicity |  |  |  |  |
| White | 1 |  | 1 |  |
| Other ethnicity | 0.86 (0.44, 1.67) | 0.651 | 0.97 (0.62, 1.50) | 0.882 |
| Gender |  |  |  |  |
| Female | 1 |  | 1 |  |
| Male | 1.64 (1.33, 2.02) | <0.001 | 1.91 (1.64, 2.22) | <0.001 |
| Townsend Deprivation Index | 1.02 (0.99, 1.06) | 0.163 | 1.01 (0.99, 1.04) | 0.435 |
| Family history of PD |  |  |  |  |
| No family history of PD | 1 |  | 1 |  |
| Family history of PD | 1.88 (1.30, 2.72) | <0.001 | 2.12 (1.65, 2.72) | <0.001 |
| Smoking status |  |  |  |  |
| Never | 1 |  | 1 |  |
| Previous | 1.00 (0.80, 1.23) | 0.977 | 0.86 (0.74, 1.00) | 0.057 |
| Current | 0.62 (0.40, 0.97) | 0.038 | 0.50 (0.36, 0.70) | <0.001 |


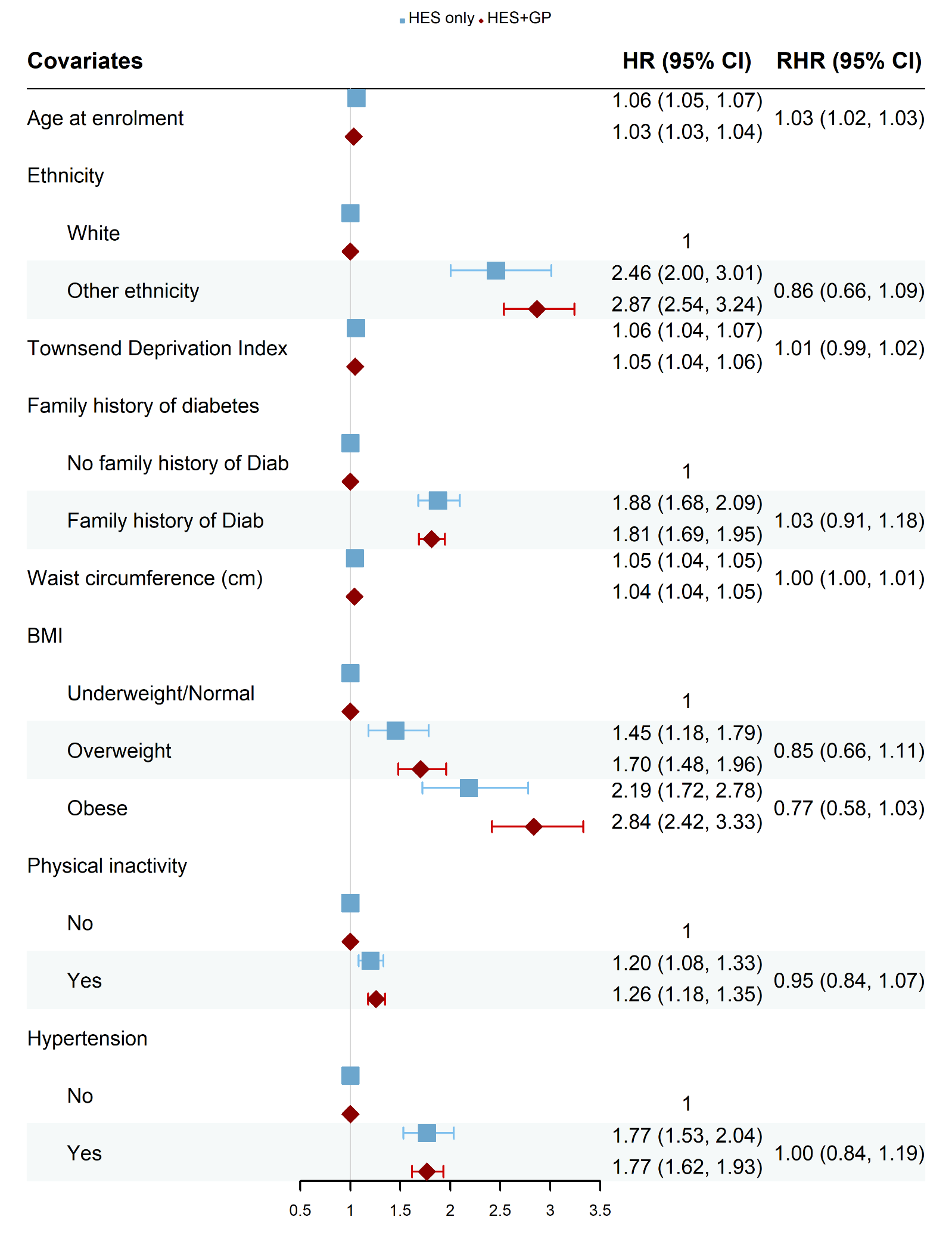


Supplementary Figure 13. Forest plot for Type 2 diabetes (Males only, complete case)

Supplementary Table 14. Complete case results of Cox models of T2D obtained using male subgroup of HES only (N=77711) and male subgroup of HES+GP population (N=77583).

|  | HES only |  | HES+GP |  |
| --- | --- | --- | --- | --- |
| Coefficient | HR (95% CI) | p | HR (95% CI) | p |
| Age at enrolment | 1.06 (1.05, 1.07) | <0.001 | 1.03 (1.03, 1.04) | <0.001 |
| Ethnicity |  |  |  |  |
| White | 1 |  | 1 |  |
| Other ethnicity | 2.46 (2.00, 3.01) | <0.001 | 2.87 (2.54, 3.24) | <0.001 |
| Townsend Deprivation Index | 1.06 (1.04, 1.07) | <0.001 | 1.05 (1.04, 1.06) | <0.001 |
| Family history of diabetes |  |  |  |  |
| No family history of Diab | 1 |  | 1 |  |
| Family history of Diab | 1.88 (1.68, 2.09) | <0.001 | 1.81 (1.69, 1.95) | <0.001 |
| Waist circumference (cm) | 1.05 (1.04, 1.05) | <0.001 | 1.04 (1.04, 1.05) | <0.001 |
| BMI |  |  |  |  |
| Underweight/Normal | 1 |  | 1 |  |
| Overweight | 1.45 (1.18, 1.79) | <0.001 | 1.70 (1.48, 1.96) | <0.001 |
| Obese | 2.19 (1.72, 2.78) | <0.001 | 2.84 (2.42, 3.33) | <0.001 |
| Physical inactivity |  |  |  |  |
| No | 1 |  | 1 |  |
| Yes | 1.20 (1.08, 1.33) | <0.001 | 1.26 (1.18, 1.35) | <0.001 |
| Hypertension |  |  |  |  |
| No | 1 |  | 1 |  |
| Yes | 1.77 (1.53, 2.04) | <0.001 | 1.77 (1.62, 1.93) | <0.001 |


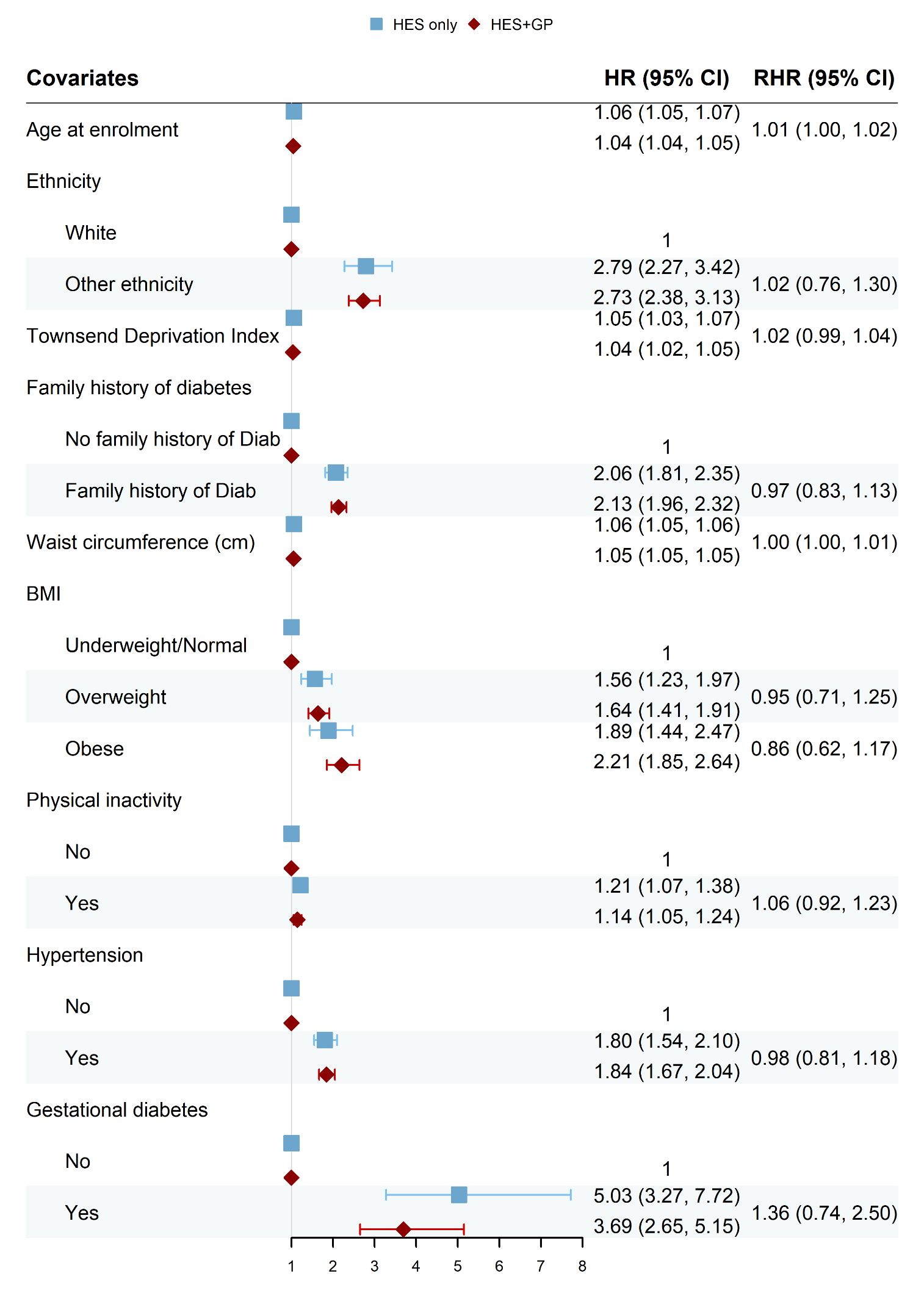


Supplementary Figure 14. Forest plot for Type 2 diabetes (Females only, complete case)

Supplementary Table 15. Complete case results of Cox models of T2D obtained using female subgroup of HES only (N=89916) and female subgroup of HES+GP population (N=89829).

|  | HES only |  | HES+GP |  |
| --- | --- | --- | --- | --- |
| Coefficient | HR (95% CI) | p | HR (95% CI) | p |
| Age at enrolment | 1.06 (1.05, 1.07) | <0.001 | 1.04 (1.04, 1.05) | <0.001 |
| Ethnicity |  |  |  |  |
| White | 1 |  | 1 |  |
| Other ethnicity | 2.79 (2.27, 3.42) | <0.001 | 2.73 (2.38, 3.13) | <0.001 |
| Townsend Deprivation Index | 1.05 (1.03, 1.07) | <0.001 | 1.04 (1.02, 1.05) | <0.001 |
| Family history of diabetes |  |  |  |  |
| No family history of Diab | 1 |  | 1 |  |
| Family history of Diab | 2.06 (1.81, 2.35) | <0.001 | 2.13 (1.96, 2.32) | <0.001 |
| Waist circumference (cm) | 1.06 (1.05, 1.06) | <0.001 | 1.05 (1.05, 1.05) | <0.001 |
| BMI |  |  |  |  |
| Underweight/Normal | 1 |  | 1 |  |
| Overweight | 1.56 (1.23, 1.97) | <0.001 | 1.64 (1.41, 1.91) | <0.001 |
| Obese | 1.89 (1.44, 2.47) | <0.001 | 2.21 (1.85, 2.64) | <0.001 |
| Physical inactivity |  |  |  |  |
| No | 1 |  | 1 |  |
| Yes | 1.21 (1.07, 1.38) | 0.003 | 1.14 (1.05, 1.24) | 0.002 |
| Hypertension |  |  |  |  |
| No | 1 |  | 1 |  |
| Yes | 1.80 (1.54, 2.10) | <0.001 | 1.84 (1.67, 2.04) | <0.001 |
| Gestational diabetes |  |  |  |  |
| No | 1 |  | 1 |  |
| Yes | 5.03 (3.27, 7.72) | <0.001 | 3.69 (2.65, 5.15) | <0.001 |


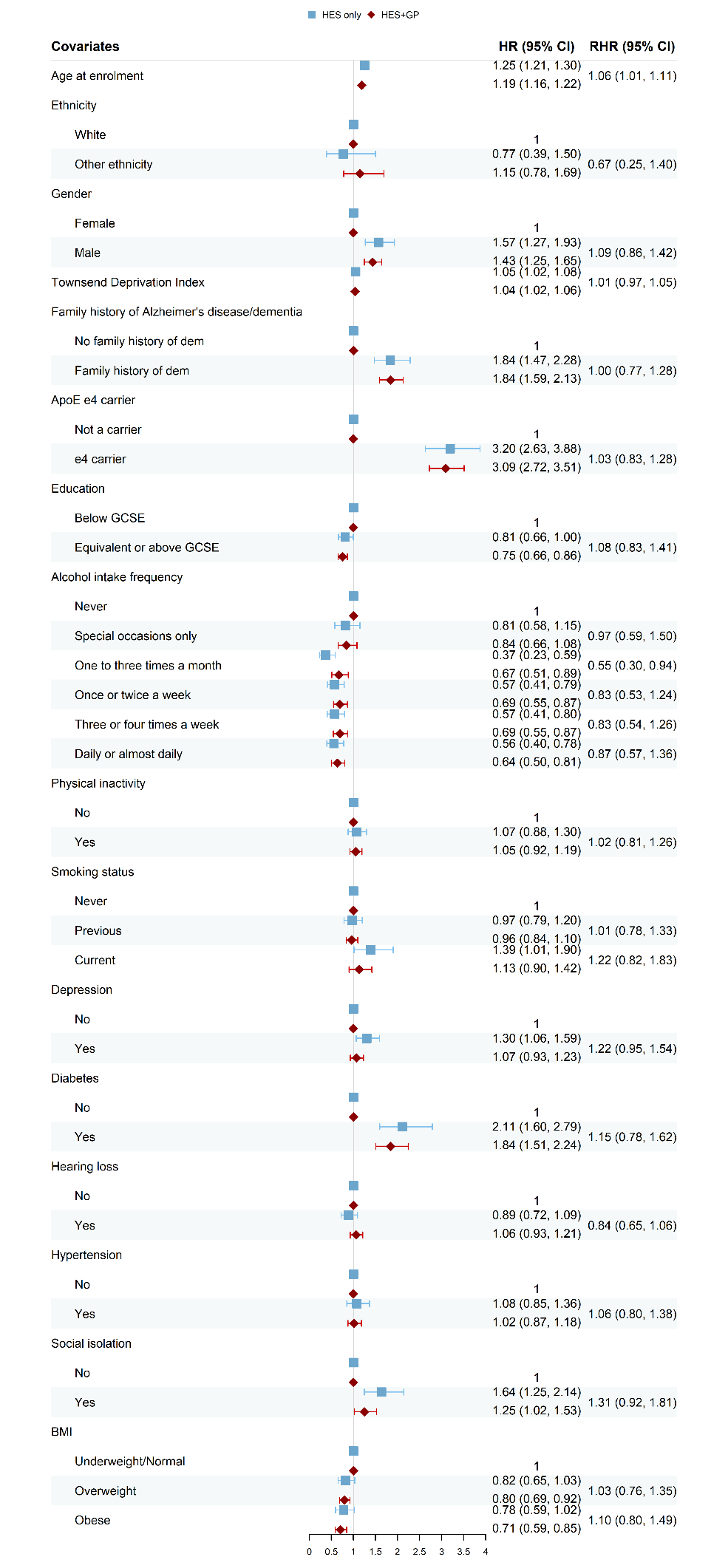


Supplementary Figure 15. Forest plot for dementia

Supplementary Table 16. Complete case results of Cox models of dementia obtained using HES only (N=69202) and HES+GP population (N=69177).

|  | HES only |  | HES+GP |  |
| --- | --- | --- | --- | --- |
| Coefficient | HR (95% CI) | p | HR (95% CI) | p |
| Age at enrolment | 1.25 (1.21, 1.30) | <0.001 | 1.19 (1.16, 1.22) | <0.001 |
| Ethnicity |  |  |  |  |
| White | 1 |  | 1 |  |
| Other ethnicity | 0.77 (0.39, 1.50) | 0.440 | 1.15 (0.78, 1.69) | 0.477 |
| Gender |  |  |  |  |
| Female | 1 |  | 1 |  |
| Male | 1.57 (1.27, 1.93) | <0.001 | 1.43 (1.25, 1.65) | <0.001 |
| Townsend Deprivation Index | 1.05 (1.02, 1.08) | 0.002 | 1.04 (1.02, 1.06) | <0.001 |
| Family history of Alzheimer’s disease/dementia |  |  |  |  |
| No family history of dem | 1 |  | 1 |  |
| Family history of dem | 1.84 (1.47, 2.28) | <0.001 | 1.84 (1.59, 2.13) | <0.001 |
| ApoE e4 carrier |  |  |  |  |
| Not a carrier | 1 |  | 1 |  |
| e4 carrier | 3.20 (2.63, 3.88) | <0.001 | 3.09 (2.72, 3.51) | <0.001 |
| Education |  |  |  |  |
| Below GCSE | 1 |  | 1 |  |
| Equivalent or above GCSE | 0.81 (0.66, 1.00) | 0.049 | 0.75 (0.66, 0.86) | <0.001 |
| Alcohol intake frequency |  |  |  |  |
| Never | 1 |  | 1 |  |
| Special occasions only | 0.81 (0.58, 1.15) | 0.241 | 0.84 (0.66, 1.08) | 0.175 |
| One to three times a month | 0.37 (0.23, 0.59) | <0.001 | 0.67 (0.51, 0.89) | 0.005 |
| Once or twice a week | 0.57 (0.41, 0.79) | <0.001 | 0.69 (0.55, 0.87) | 0.001 |
| Three or four times a week | 0.57 (0.41, 0.80) | 0.001 | 0.69 (0.55, 0.87) | 0.002 |
| Daily or almost daily | 0.56 (0.40, 0.78) | <0.001 | 0.64 (0.50, 0.81) | <0.001 |
| Physical inactivity |  |  |  |  |
| No | 1 |  | 1 |  |
| Yes | 1.07 (0.88, 1.30) | 0.506 | 1.05 (0.92, 1.19) | 0.452 |
| Smoking status |  |  |  |  |
| Never | 1 |  | 1 |  |
| Previous | 0.97 (0.79, 1.20) | 0.805 | 0.96 (0.84, 1.10) | 0.572 |
| Current | 1.39 (1.01, 1.90) | 0.042 | 1.13 (0.90, 1.42) | 0.278 |
| Depression |  |  |  |  |
| No | 1 |  | 1 |  |
| Yes | 1.30 (1.06, 1.59) | 0.010 | 1.07 (0.93, 1.23) | 0.335 |
| Diabetes |  |  |  |  |
| No | 1 |  | 1 |  |
| Yes | 2.11 (1.60, 2.79) | <0.001 | 1.84 (1.51, 2.24) | <0.001 |
| Hearing loss |  |  |  |  |
| No | 1 |  | 1 |  |
| Yes | 0.89 (0.72, 1.09) | 0.263 | 1.06 (0.93, 1.21) | 0.394 |
| Hypertension |  |  |  |  |
| No | 1 |  | 1 |  |
| Yes | 1.08 (0.85, 1.36) | 0.540 | 1.02 (0.87, 1.18) | 0.829 |
| Social isolation |  |  |  |  |
| No | 1 |  | 1 |  |
| Yes | 1.64 (1.25, 2.14) | <0.001 | 1.25 (1.02, 1.53) | 0.030 |
| BMI |  |  |  |  |
| Underweight/Normal | 1 |  | 1 |  |
| Overweight | 0.82 (0.65, 1.03) | 0.089 | 0.80 (0.69, 0.92) | 0.003 |
| Obese | 0.78 (0.59, 1.02) | 0.070 | 0.71 (0.59, 0.85) | <0.001 |


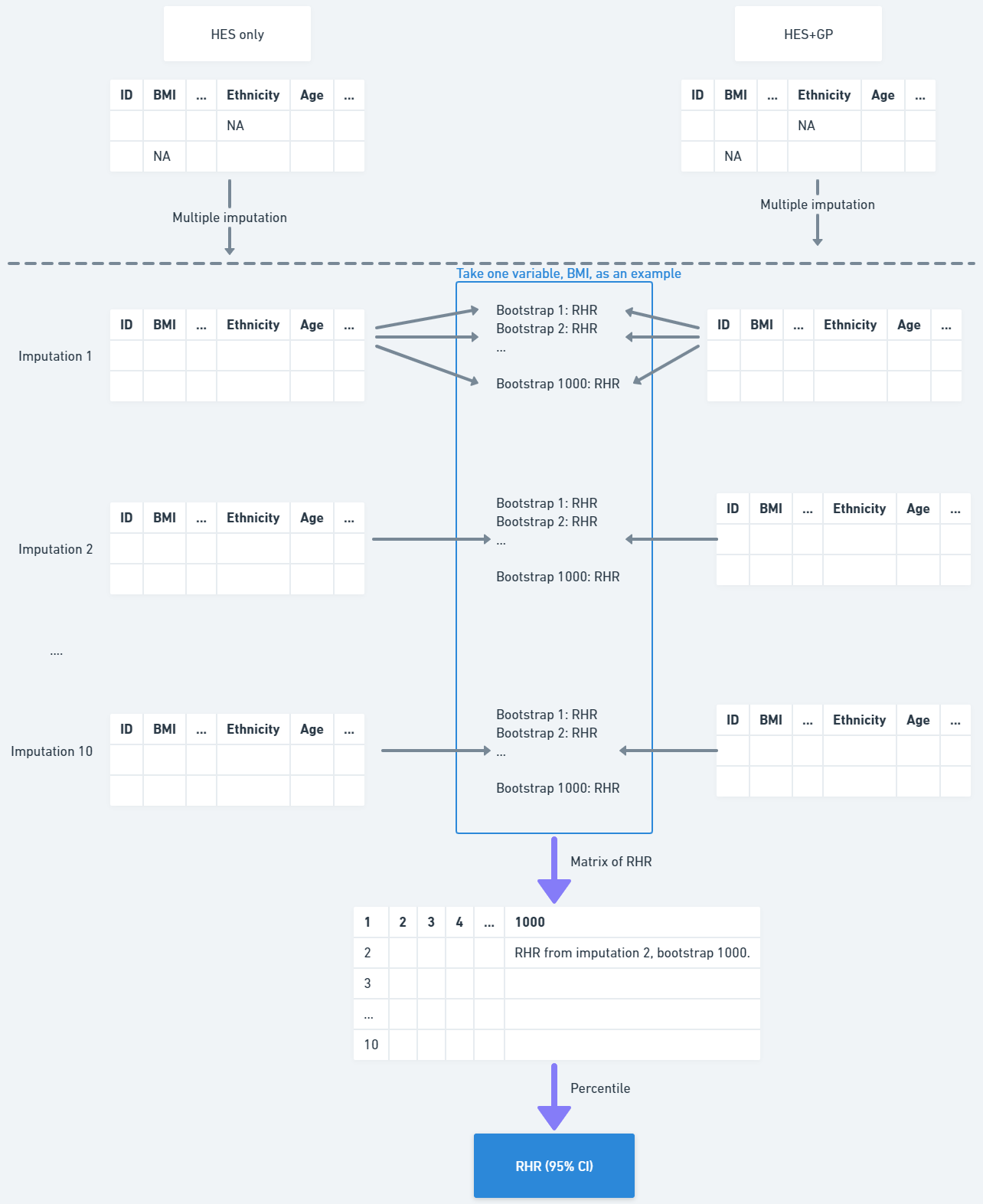


Supplementary Figure 16. Illustration of multiple imputation bootstrap (pooled sample) approach from [Schomaker2018](https://onlinelibrary.wiley.com/doi/10.1002/sim.7654). RHR: ratio of hazard ratio.
